# pVHL regulates protein stability of the TCF/LEF transcription factor family via ubiquitin-independent proteasomal degradation

**DOI:** 10.1007/s00018-025-05852-0

**Published:** 2025-09-04

**Authors:** Caixia Wang, Xiaozhi Rong, Haifeng Zhang, Bo Wang, Yan Bai, Yonghua Sun, Chengtian Zhao, Jianfeng Zhou

**Affiliations:** 1 Key Laboratory of Marine Drugs (Ocean University of China), Chinese Ministry of Education, and School of Medicine and Pharmacy, Ocean University of China, 5 Yushan Road, Qingdao, 266003 China; 2 Laboratory for Marine Drugs and Bioproducts, Qingdao Marine Science and Technology Center, Qingdao, 266237 China; 3 State Key Laboratory of Freshwater Ecology and Biotechnology, Institute of Hydrobiology, Innovation Academy for Seed Design, Chinese Academy of Sciences, Wuhan, 430072 China; 4 College of Advanced Agricultural Sciences, University of Chinese Academy of Sciences, Beijing, 100049 China; 5 Institute of Evolution and Marine Biodiversity and College of Marine Biology, Ocean University of China, 5 Yushan Road, Qingdao, 266003 China; 6 Laboratory for Marine Biology and Biotechnology, Qingdao Marine Science and Technology Center, Qingdao, 266237 China

**Keywords:** pVHL, TCF/LEF, Ubiquitin-independent proteasomal degradation, Wnt/β-catenin signaling, Dorsal habenular neurons

## Abstract

The Wnt/β-catenin signaling pathway plays key roles in development and adult tissue homeostasis by controlling cell proliferation and cell fate decisions. TCF/LEF transcription factors play a pivotal role in this pathway, acting as repressors by recruiting co-repressors in the absence of Wnt signals, and as activators via β-catenin binding in the presence of Wnt signaling. While progress has been made in our understanding of Wnt signaling regulation, the underlying mechanism that regulates the protein stability of the TCF/LEF family is far less clear. Using cultured cells and zebrafish as *in vitro* and *in vivo* models, we demonstrated that the von Hippel-Lindau protein (pVHL), the substrate recognition component of an E3 ubiquitin ligase complex, regulates the stability of TCF/LEF proteins. Unexpectedly, pVHL directly binds to TCF/LEF and promotes their proteasomal degradation independent of its E3 ubiquitin ligase activity. Notably, a human homologue of pVHL, the pVHL-like protein (pVHLL), which lacks the capability to assemble an E3 ligase complex with Elongin B/C, RBX1, and CUL2, similarly downregulates TCF/LEF protein levels. Knockout of *vhl* in zebrafish embryos leads to a reduction of dorsal habenular neurons and this effect is upstream of dorsal habenular neurons phenotype in *tcf7l2*-null mutants. Our study uncovers a previously unknown mechanism for the protein stability regulation of TCF/LEF transcription factors and demonstrates that pVHL contains a 26S proteasome binding domain that drives ubiquitin-independent proteasomal degradation. These findings provide new insights into the ubiquitin-independent function of pVHL and uncover a novel mechanistic regulation of Wnt/β-catenin signaling.

**Supplementary Information:**

The online version contains supplementary material available at 10.1007/s00018-025-05852-0.

## Introduction

The Wnt/β-catenin signaling pathway is an evolutionarily conserved signal transduction cascade that controls numerous developmental processes and plays crucial roles in the regulation of diverse processes, including stem cell renewal, cell proliferation, and cell differentiation during adult tissue homeostasis in multicellular animals [1–5]. Dysregulation of the Wnt/β-catenin signaling cascade is often associated with various kinds of human diseases, including many cancers and hereditary diseases [1, 3, 4, 6]. In the absence of Wnt ligands, cytosolic β-catenin interacts with a destruction complex consisting of adenomatous polyposis coli (APC), glycogen synthase kinase 3 (GSK3), casein kinase 1 (CK1), axis inhibition protein 1/2 (AXIN1/2) which leads to the phosphorylation of N-terminal Ser/Thr residues of β-catenin by CK1 and GSK3. Consequently, β-catenin is ubiquitylated and undergoes proteasome-mediated degradation to maintain the cytoplasmic β-catenin at low levels [1, 3, 7]. Once Wnt ligands bind to the Frizzled family transmembrane receptors and LRP5/6 coreceptors, the disaggregation of the destruction complex is triggered. Consequently, β-catenin is non-phosphorylated and stabilized, which allows it to accumulate in the cytoplasm and translocate into the nucleus. In the nucleus, DNA-bound T-cell factor/lymphoid enhancer-binding factor (TCF/LEF) transcription factors act as transcriptional repressors by interacting with Groucho proteins, while they can transiently convert into transcriptional activators upon engagement with β-catenin [1, 3, 4]. Thus, the ultimate outcome of the Wnt signal is determined by β-catenin and the TCF/LEF.

All members of the TCF/LEF family are high-mobility group (HMG) DNA-binding proteins which contain a conserved HMG box domain responsible for sequence-specific DNA recognition and binding, a nuclear localization signal (NLS), as well as multiple domains that mediate protein interactions and regulations [1, 3, 8, 9]. To date, four TCF/LEF protein family members have been identified in vertebrate genomes. Currently available evidence suggests that the relative amounts of β-catenin and TCF/LEF in the nucleus influence the Wnt signaling output [10, 11]. Hence, nuclear TCF/LEF concentrations may be dynamically controlled as precisely as that of β-catenin [9]. Compared with the well understood protein stability regulation of β-catenin as an effector of the Wnt signaling pathway, however, the stability of the TCF/LEF proteins is far less clear.

The von Hippel-Lindau protein (pVHL) is the target protein recognition subunit of an E3 ubiquitin ligase complex (VBC, including pVHL and Elongins B and C) [12]. Various natural mutations with inactivation of the *VHL* gene, a tumor suppressor gene, have been reported in most cases of hereditary von Hippel-Lindau disease and sporadic clear cell renal cell carcinomas (ccRCCs) [12, 13]. pVHL targets prolyl-hydroxylated proteins [14–17]. Hypoxia-inducible factors (HIF-α), which are prolyl-hydroxylated by EGLN 1/2/3 family proteins under normal oxygen tension, are the well-documented canonical targets of pVHL. Hydroxylated HIF-α is bound and recognized by pVHL for ubiquitination-mediated proteasomal degradation [15, 16]. In contrast, under hypoxic or pVHL-deficient condition, HIF-1α is constitutively degraded by proteasome via a mechanism that is independent of oxygen, pVHL, and ubiquitylation [18].

We previously reported that VBP1, a pVHL binding protein, promotes the proteasomal degradation of TCF/LEF through pVHL [19]. In this process, pVHL is essential for VBP1-mediated regulation of TCF/LEF stability. Intriguingly, we observed that VBP1 enhances the ubiquitylation of HIF-1α but fails to induce the ubiquitylation of Tcf7l2. These findings suggest that pVHL likely promotes the proteasomal degradation of TCF/LEF through a previously unrecognized mechanism. In the present study, we aim to elucidate the molecular mechanisms by which pVHL regulates TCF/LEF protein stability and to investigate the physiological significance of this interaction using a model organism.

## Materials and methods

### Cell lines, chemicals, reagents, and antibodies

HEK293T, HeLa, HCT116, and 786-O cell lines were purchased from the American Type Culture Collection (ATCC; Manassas, VA, USA). Dulbecco’s modified Eagle’s medium (DMEM, SH30243.01), Roswell Park Memorial Institute medium (RPMI, SH30809.01), and penicillin/streptomycin (SV30010) were purchased from Hyclone (Logan, UT, USA). Fetal bovine serum (FBS) was purchased from PAN (ST30-3302, Aidenbach, Germany). TAK243 (S8341) and MG132 (S2619) was purchased from SelleckChem (Houston, TX, USA). NH_4_Cl (A9434) and 3-Methyladenine (3-MA, M9281) was purchased from Sigma (St. Louis, MO, USA). Dimethyloxallyl Glycine (DMOG, HY-15893) was purchased from MedChemExpress (Monmouth, NJ, USA). The polyethylenimine (#23966–2) was purchased from Polysciences Inc (Warrington, PA, USA), and the Lipofectamine RNAiMAX (#13778100) was purchased from Invitrogen (Carlsbad, CA, USA). The protease inhibitor cocktail mixture (C0001) was purchased from TargetMol (Boston, MA, USA). Adenosine 5'-triphosphate (ATP, A2383), creatine phosphate (P7936), and creatine kinase (C3755) was purchased from Sigma. DAPI (C1002) and puromycin (ST551) were purchased from Beyotime (Shanghai, China). Glutathione Sepharose 4B beads (71024800-GE) was purchased from GE Healthcare (Chicago, IL, USA). Protein A/G Plus-agarose (sc-2003) was purchased from Santa Cruz Biotechnology (Dallas, TX, USA). TRIzol reagent (15596018CN) was purchased from Invitrogen. M-MLV (# 9PIM170) was purchased from Promega (Madison, WI, USA). The information regarding primers and antibodies is provided in Supplementary files 12 and 13, respectively.

### Molecular cloning and plasmid construction

The expression plasmids pCS2-6 × Myc-Tcf7, pCS2-6 × Myc-Tcf7l1, pCS2-6 × Myc-Tcf7l2, and pCS2-6 × Myc-Lef1, which have a *Xenopus* background, were kindly provided by Dr. Wei Wu (School of Life Sciences, Tsinghua University). The expression plasmids pCS2-Flag-pVHL, pCS2-Myc-pVHL, pGEX-2 T-pVHL, pBoBi-puro-Flag-pVHL, pCS2-pVHL-GFP, pCS2-pVHL-P2A-GFP, pCS2-Flag-pVHLL, pCDNA3.1-TCF7L2-HA, pCS2-Flag-HIF-1α, and various pVHL or TCF7L2 mutants with a human background were constructed via PCR. Expression vector pGEX-2 T (28–9546-53) was purchased from GE Healthcare. Plasmid Super 8 × TOPFlash reporter was a gift from Dr. Randall Moon (Department of Pharmacology, University of Washington School of Medicine, Seattle) (Addgene plasmid #12456).

### Zebrafish strains

Zebrafish (*Danio rerio*) Tübingen wild-type (WT), the transgenic line *Tg*(*huc*:*GFP*), and the *vhl*-null and *tcf7l2*-null strains were maintained on a 14 h light/10 h dark cycle at 28.5 °C and fed twice daily. The zebrafish *vhl* mutant strain was gifted by Dr. Wuhan Xiao [20]. The zebrafish *tcf7l2* mutant allele (*tcf7l2*^+/ihb316^, ZFIN ID: ZDB-ALT-181129–11) was generated by the CRISPR/Cas9 method and obtained from the China Zebrafish Resource Center, National Aquatic Biological Resource Center (CZRC/NABRC), Wuhan, China. The animals were raised and maintained according to standard procedures described in Zebrafish Information Network (ZFIN; https://zfin.org/). Embryos obtained by natural crosses were maintained in embryo rearing solution (5 mM NaCl, 0.17 mM KCl, 0.33 mM CaCl_2_, and 0.66 mM MgSO_4_) in an incubator at 28.5 °C. The embryos were staged according to standard methods [21].

### Cell culture and transfections

HEK293T, HeLa, and HCT116 cells were cultured in DMEM supplemented with 10% FBS and 1% penicillin/streptomycin at 37 ℃ in 5% CO_2_. The 786-O cells were cultured in RPMI medium supplemented with 10% FBS and 1% penicillin/streptomycin at 37 ℃ in 5% CO_2_. The cell lines underwent short tandem repeat profiling analysis conducted by ShCellBank (Shanghai, China) to confirm their identity. Additionally, to ensure free of mycoplasma contamination, we performed test every three months by using the EZ-PCR Mycoplasmas Detection Kit (BI, Kibbutz Beit-Haemek, Israel).

Hypoxia-treated cells were performed in a hypoxic chamber containing 1% O_2_ at 37 ℃ for 24 h as reported previously [22]. For starvation treatment, HEK293T cells were washed twice with DMEM and cultured under serum starvation in a time series. In some experiments, cells were treated with MG132 (10 μM), NH_4_Cl (25 mM), or 3-MA (5 mM) for 8 h to inhibit proteasome activity, lysosomal function, or autophagy. TAK243 (1 μM) was used to inhibit the ubiquitin activating enzyme for 12 h in HEK293T cells. DMOG (200 μM) was added to the culture medium of HEK293T cells for 12 h to inhibit prolyl hydroxylase activity.

Plasmid transfection was carried out using polyethylenimine according to the manufacturer's instructions. The siRNAs targeting *ELOC* were synthesized by GenePharma (Shanghai, China) with the sequences 5'-CUAUCGAAAGUAUGCAUGU-3'and 5'-CGAACUUCUUAGAUUGUUA-3'. HEK293T cells were transfected with these siRNAs using Lipofectamine RNAiMAX following the manufacturer’s protocol. After 72 h of transfection, protein levels were analyzed by immunoblotting. To establish Flag-pVHL stable cell lines, lentiviral particles expressing VHL cDNA were generated and subsequently used to infect the 786-O cell line. Following viral infection, cells were selected and maintained in medium supplemented with puromycin (1 μg/mL). The overexpression efficiency of Flag-pVHL was confirmed by immunoblotting analysis.

### GST fusion protein purification and in vitro GST pulldown assays

The GST-pVHL fusion protein and its mutants, including GST-pVHL(54–99), GST-pVHL(100–157), and GST-pVHL(54–157), were expressed in the *Escherichia coli* BL21 strain. The fermentation process was initiated with a 1% inoculum and incubated at 37 °C under agitation at 150 rpm until the OD600 reached 0.6–0.8. The fusion proteins were induced by adding 1 mM IPTG and incubating at 37 °C for 5 h. Cells were harvested via centrifugation, washed with ice-cold phosphate-buffered saline (PBS; 137 mM NaCl, 2.7 mM KCl, 10 mM Na_2_HPO_4_, 1.8 mM KH_2_PO_4_), and lysed in lysis buffer (50 mM Tris–HCl, pH 7.5, 150 mM NaCl, 1 mM EDTA, 10% glycerol, 1% Triton X-100) on ice. Lysis was facilitated by sonication for a total of 30 min using a VCX 130 Sonicator (Sonics & Materials, Inc., Newtown, CT, USA) with pulse settings of 2 s on/9 s off at 40% amplitude. The GST-tagged fusion proteins were purified by mixing the cell lysates with glutathione Sepharose 4B beads overnight at 4 °C. The mixtures were subsequently washed three times with ice-cold PBS to purified GST-tagged fusion proteins.

To assess the purity and expression levels of the fusion proteins, a portion of the purified proteins was eluted with 5 × SDS/PAGE loading buffer (200 mM Tris–HCl, pH 7.0, 15% SDS, 30% glycerol, 0.05% bromophenol blue, 10% β-mercaptoethanol) by boiling for 5 min and analyzed by Coomassie blue staining. To investigate the direct interactions between pVHL and TCF as well as between pVHL and the 26S proteasome, the purified GST or GST-fusion proteins were incubated with equal volumes of HEK293T cell lysates containing the indicated components. The mixtures were rotated overnight at 4 °C, followed by extensive washing with lysis buffer. The bound proteins were eluted with SDS/PAGE loading buffer and detected using the specified antibodies.

For the interaction analysis between pVHL and the proteasome, the GST or GST-fusion protein was incubated with purified human 26S proteasome (E365; Boston Biochem, Cambridge, MA, USA) in binding buffer (1 × PBS, 2 mM EDTA, 1 mM phenylmethylsulfonyl fluoride, 0.5% Triton X-100) at 4 °C for 2 h. After washing three times with ice-cold PBS, the bound proteins were analyzed by western blot.

### Surface plasmon resonance (SPR) analysis

The recombinant proteins for human pVHL (CSB-EP025852HU) and TCF7L2(1–456) (CSB-EP889079HU), expressed in the *Escherichia coli* expression system, were obtained from CUSABIO (Wuhan, China). Surface plasmon resonance (SPR) experiments to analyze the interaction between pVHL and TCF7L2(1–456) were performed using a BIAcore T200 system (GE Healthcare). A series S CM5 sensor chip was employed, with channel one used for immobilizing pVHL protein via amine coupling using pH 4.5 sodium acetate buffer (GE Healthcare), while channel two was treated identically but without pVHL as a reference. The analyte TCF7L2(1–456) was prepared at concentrations of 0.36 μM, 0.73 μM, 1.09 μM, 1.46 μM, and 1.82 μM in PBS. PBS buffer served as the running buffer at a flow rate of 10 μL/min. The analyte was injected over the sensor surface for 60 s, followed by a dissociation phase lasting 60 s. Sensor regeneration was achieved using 2.5 mM NaOH for 15 s. Steady-state affinity analysis of the interaction between pVHL and TCF7L2(1–456) was conducted using Biacore T200 evaluation software version 3.1.

### Luciferase assays

The cells were transfected with the TOPFlash reporter plasmid to evaluate Wnt/β-catenin signaling activity. Transfection efficiency was normalized by co-transfecting a *Renilla* luciferase reporter plasmid. Cells were lysed using 1 × passive lysis buffer (E1960, Promega), and the TOPFlash/*Renilla* luciferase activities were measured using the Dual-Luciferase Reporter Assay System (E1960, Promega) according to the manufacturer's instructions.

### Cycloheximide chase assay

HEK293T cells were transfected with either an empty vector, Myc-pVHL, or Flag-pVHL (1–157). After 48 h, the cells were treated with cycloheximide (CHX; 100 μg/mL) and harvested at the specified time points (0, 2, 4, 8 h). Cell lysates were prepared and subjected to immunoblot analysis. Protein stability was quantified by determining the percentage of TCF7L2E or TCF7L2M/S remaining relative to the initial time point after normalization to Histone H3 levels.

### Ubiquitination assay

Ubiquitination assays were conducted using hot lysis-extracted protein lysates, following a previously described protocol [23]. Briefly, HEK293T cells were co-transfected with Myc-Tcf7l2, Flag-HIF-1α, and HA-Ub plasmids, along with either GFP or pVHL-GFP. Cells were treated with 10 μM MG132 for 8 h prior to harvest. Hot lysis was performed by boiling the cells in 100 µL denaturing buffer (2% SDS, 10 mM Tris–HCl, pH 8.0, 150 mM NaCl, and 1 × protease inhibitor cocktail supplemented with 10 mM freshly prepared N-ethylmaleimide, a deubiquitinating enzyme inhibitor) for 10 min. The lysates were subsequently diluted ten-fold with dilution buffer (1% Triton X-100, 10 mM Tris–HCl, pH 8.0, 150 mM NaCl, 2 mM EDTA, and 1 × protease inhibitor cocktail with 10 mM freshly prepared N-ethylmaleimide). Tcf7l2 and HIF-1α proteins were immunoprecipitated from whole-cell lysates using specific antibodies in conjunction with Protein A/G resin. Ubiquitinated forms of Tcf7l2 and HIF-1α were detected by immunoblot analysis using the indicated antibodies.

### Degradation assay in cell-free system

To analyze the degradation rate of the target protein, a cell-free system was employed by incubating the purified target protein with the 26S proteasome [18, 24]. Specifically, 1 μg of TCF7L2(1–456) was incubated with 2 μg of purified 26S proteasome (PS026, LifeSensors, Malvern, USA) in a reaction buffer containing 50 mM Tris–HCl (pH 7.5), 100 mM KCl, 10% glycerol, 10 mM MgCl_2_, 1 mM DTT, 0.1 mM EDTA, 2 mM ATP, 10 mM creatine phosphate, and 30 μg/mL creatine kinase, with a final reaction volume of 20 μL. The mixture was incubated at 37 °C, and 2 μL aliquots were collected at 0, 2, 4, 6, 12, and 18 h for subsequent analysis. To confirm the specificity of 26S proteasome-mediated degradation of the target protein, a control group treated with MG132 was included.

### SDS/PAGE and Coomassie blue staining

Protein samples were loaded onto the gel according to the experimental requirements, and electrophoresis was performed at 80 V for 30 min, followed by 120 V for 30 to 60 min. After electrophoresis, the gel was rinsed with deionized water and stained with BeyoBlue™ Plus Coomassie Blue SuperFast Staining Solution (P0003S, Beyotime) for 30 min. The gel was subsequently destained by washing with deionized water, with the water being replaced every 30 min until the target band became clearly visible and the background was clean.

### Western blot

Cells or zebrafish embryos were lysed in RIPA buffer (150 mM NaCl, 1% Triton X-100, 1% sodium deoxycholate, 0.1% SDS, and 50 mM Tris–HCl at pH 7.5) supplemented with protease inhibitors on ice for 15 min. Subsequently, the samples were centrifuged at 14,000 × g for 10 min at 4 °C, and the supernatant was collected. Finally, the samples were boiled for 5 min in SDS/PAGE loading buffer. Protein samples were then separated by SDS-PAGE and transferred to PVDF membranes using a semi-dry electrophoretic transfer system (Bio-Rad Laboratories, Inc., Hercules, CA, USA) under constant current for 1.5 h. The protein bands were visualized using BeyoECL Plus reagent (P0018M, Beyotime).

### Immunoprecipitation (Co-IP) and mass spectrometry analysis

For the Co-IP experiments, cells were lysed in IP lysis buffer containing 50 mM Tris–HCl (pH 7.5), 150 mM NaCl, 1 mM EDTA, 10% glycerol, 1% Triton X-100, and protease inhibitors. Cleared cell lysates were incubated with the appropriate antibodies (1–2 μg) overnight at 4 °C, followed by a 4 h incubation at 4 °C with Protein A/G agarose beads. The immune complexes bound to the Protein A/G beads were washed five times with IP lysis buffer and eluted with SDS loading buffer for subsequent immunoblot analysis. At least three independent experiments were performed.

For mass spectrometry analysis, HEK293T cells were transfected with either pCS2-Flag or pCS2-Flag-pVHL expression vectors for 48 h. Prior to harvest, the cells were treated with the proteasome inhibitor MG132 (10 μM) for 8 h. Following washing and elution according to the immunoprecipitation protocol, the samples were subjected to Coomassie Blue staining and analyzed by liquid chromatography-mass spectrometry (LC–MS/MS) using a nanoElute UHPLC system coupled to a timsTOF Pro 2 mass spectrometer (Bruker Daltonics, Bilerica, MA, USA). Data acquisition was performed in data-independent acquisition (DIA) mode, and the resulting data were processed using Spectronaut software (Biognosys, Zurich, Switzerland). The Human UniProt SwissProt proteome database (release 2024_07_26, containing 20,436 protein sequences) was used for peptide identification.

### Immunofluorescence staining

Cells were cultured on coverslips. Following 24 h of transfection, the cells were fixed with 4% paraformaldehyde for 15 min at room temperature, permeabilized with PBS containing 0.2% Triton X-100 for 5 min at room temperature, and blocked with 3% bovine serum albumin (BSA) in PBS for 1 h at room temperature. The cells were then incubated with primary antibodies overnight at 4 °C. After three washes with PBS, the cells were incubated with secondary antibodies in the dark for 2 h at room temperature. Subsequently, the cells were stained with DAPI (1:5000) in PBS for 15 min, mounted in 50% glycerol. Confocal images were acquired using a Leica SP8 confocal microscope (Leica Microsystems, Wetzlar, Germany).

### EdU (5-Ethynyl-2’-deoxyuridine) assay

The EdU assay was performed using the Click-iT EdU Alexa Fluor® 488 Cell Proliferation Assay Kit (C10337, Invitrogen). At 48 h post-transfection, HCT116 cells were incubated with 10 μM EdU in fresh culture medium for 2 h. Subsequently, the cells were fixed with 4% formaldehyde for 15 min at room temperature and permeabilized with 0.5% Triton X-100 in PBS for 10 min. The cells were then incubated with the Click-iT reaction cocktail for 30 min in the dark at room temperature. Following this, the cells were washed three times with PBS containing 3% BSA. Nuclei were counterstained with DAPI (diluted 1:5000).

### Generation of knockout cell lines via CRISPR/Cas9

The gRNAs were designed using the CRISPR V2 tool (http://zifit.partners.org). The gRNA target site 1 (Tg1, 5′- GCGCGCGCGAAGACTACGG-3′) located before 5’ UTR, and the gRNA target site 2 (Tg2, 5′-CGCGTCGTGCTGCCCGTAT-3′) located within the first exon, were selected as the CRISPR targeting sites for *VHL* in the human cell line. The target sequence for HIF1-β (5′-TGAAATTGAACGGCGGCGA-3′), located within the sixth exon. For CRISPR-mediated knockout, cells were transfected with plasmids expressing the specified gRNA and Cas9, and subsequently selected using antibiotics.

### Quantitative real-time reverse transcription PCR (qRT-PCR)

Total RNA was isolated from cultured cells using TRIzol reagent. The cDNAs were reverse-transcribed into first-strand cDNA using Oligo(dT)_18_ and M-MLV according to the manufacturer’s instructions. The qRT-PCR was performed using an iCycler iQ Multicolor Real-time PCR Detection System (Bio-Rad Laboratories). Primer sequences used for the qRT-PCR experiments are provided in Supplementary file 12. Samples from three independent experiments were collected, and each sample was measured in duplicate. The mRNA levels of the genes of interest were calculated using the 2^−∆∆Ct^ method and normalized to *β-actin*.

### Chromatin immunoprecipitation (ChIP) assays

ChIP assays were performed as previously described [25]. WT or *VHL*-knockout HEK293T cells (2 × 10^7) were crosslinked with freshly prepared 1% formaldehyde. Genomic DNA was sheared into fragments ranging from 200 to 1500 bp using a VCX 130 sonicator (3 min total duration, 10 s on/10 s off pulses at 40% amplitude). Cell lysates were immunoprecipitated with an anti-TCF7L2 antibody overnight at 4 °C. Immune complexes were subsequently captured by incubation with Protein A/G agarose beads for 1 h at 4 °C. The immune complexes bound to the Protein A/G beads were sequentially washed at 4 °C with the following buffers: low-salt immune complex wash buffer (20 mM Tris–HCl, pH 8.0, 2 mM EDTA, 150 mM NaCl, 1% Triton X-100, and 0.1% SDS), high-salt immune complex wash buffer (20 mM Tris–HCl, pH 8.0, 2 mM EDTA, 500 mM NaCl, 1% Triton X-100, and 0.1% SDS), LiCl immune complex wash buffer (10 mM Tris–HCl, pH 8.0, 1 mM EDTA, 250 mM LiCl, 1% NP-40, and 1% SDS), and TE buffer (10 mM Tris–HCl, pH 8.0, 1 mM EDTA). Immune complexes were eluted at 65 °C with elution buffer (25 mM Tris–HCl, pH 8.0, 10 mM EDTA, and 0.5% SDS). Crosslinks were reversed at 65 °C overnight, followed by RNA and protein removal using RNase A and Proteinase K treatment. DNA was then extracted, purified, and analyzed by quantitative real-time PCR (qPCR). Primer sequences used for qPCR are listed in Supplementary flie 12. Samples from three independent experiments were collected, and each sample was measured in duplicate.

### Microinjection

The capped mRNAs were generated *in vitro* with the mMESSAGE mMACHINE Kit (Ambion, Austin, TX, USA). Diluted mRNA was injected into 1–2 cell stage zebrafish embryos. The phenotype of embryos was observed at 12.5 h post fertilization (hpf) or 24 hpf. Expression pattern of dorsal markers *gsc* and *chd* was detected at 4.3 hpf. The protein expression of GFP in zebrafish embryos was detected at 6 hpf.

### Whole-mount in situ hybridization and immunohistochemical staining

Whole-mount *in situ* hybridization using a digoxigenin-labeled RNA riboprobe was performed as previously described [26]. For fluorescent *in situ* hybridization, a fluorescein-labeled RNA riboprobe was used, followed by incubation with an anti-fluorescein-POD antibody (11426346910, Roche, Basel, Switzerland) and signal amplification with TSA plus Fluorescein or Cyanine 3 detection kit (NEL741001KT or NEL744001KT, PerkinElmer, Waltham, Massachusetts, USA).

Antibody staining was performed according to standard procedures [27]. Embryos were fixed with 4% formaldehyde at the indicated time points and washed with PBST containing 0.8% Triton X-100 in PBS. Embryos at 36–48 hpf were treated with proteinase K for 30 min, followed by re-fixation with 4% formaldehyde for 20 min. After blocking for at least 1 h, embryos were incubated with the primary antibody overnight at 4 °C. Following extensive washing with PBST, embryos were incubated with the secondary antibody overnight at 4 °C. Finally, nuclei were stained with DAPI (1:5000).

### Microscopy and image analysis

Stained embryos were mounted in 100% glycerol, while live embryos were immobilized in 2% methylcellulose for imaging. Images were acquired using a Leica M205 FCA microscope. Quantitative analysis of the *kctd12.1* expression domain on the left side was performed using ImageJ software.

Confocal imaging was performed using a Leica TCS SP8 STED microscope. For imaging, *Tg(huc:GFP)* embryos or whole-mount immunohistochemically stained embryos were mounted in 1.2% low-melt agarose in glass-bottom dishes. Images were acquired using a 40 × water-immersion objective lens. To quantify the number of neuronal cells in dorsal habenula (dHb), we utilized the transgenic line *Tg(huc:GFP)* in combination with nuclear DAPI staining (1:5000). The numbers of HuC:GFP^+^ neurons on the left and right sides were counted from confocal z-stacks acquired at 2 μm intervals using ImageJ software.

### Statistical analysis

Graphs were generated with GraphPad Prism 9 Software (GraphPad Software, La Jolla, CA, USA). Statistical analyses were conducted using different methods, depending on the comparison being made. A two-tailed, unpaired Student’s *t*-test was used for comparing two groups, while one-way ANOVA followed by Tukey’s or Dunnett's post-hoc tests was employed for comparisons among multiple groups. Additionally, two-way ANOVA followed by Bonferroni's post-hoc test was utilized when two independent variables influence a dependent variable. *p* < 0.05 or smaller *p* value was considered statistically significant. Unless otherwise indicated, all experiments were performed in triplicate, and the data were presented as means ± S.D. for three independent experiments.

## Results

### pVHL inhibits Wnt/β-catenin signaling and promotes TCF/LEF protein degradation

**Fig. 1:**
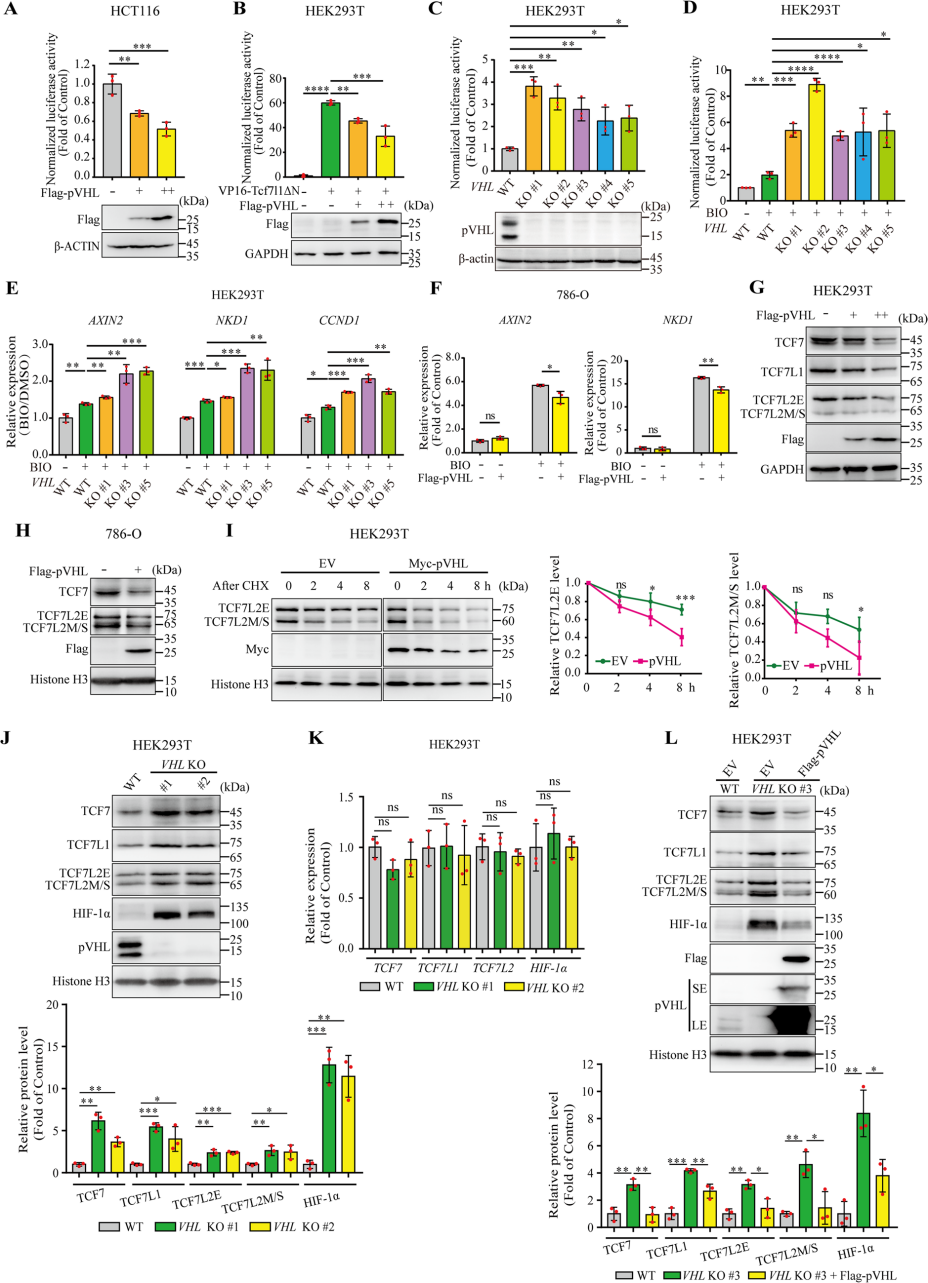
pVHL inhibits Wnt/β-catenin signaling and destabilizes TCF/LEF protein (**A**) TOPFlash luciferase assays in HCT116 cells with increasing pVHL overexpression. Expression of Flag-pVHL was confirmed by western blotting. Values are mean ± S.D. (*n* = 3). One-way ANOVA analysis with Dunnett's multiple comparisons test, **p < 0.01; ***p < 0.001. (**B**) TOPFlash assays in VP16-Tcf7l1ΔN-treated HEK293T cells with increasing pVHL overexpression. Expression of Flag-pVHL was confirmed by western blotting. Wnt/β-catenin signal was activated by transfection with VP16-Tcf7l1ΔN plasmid DNA (50 ng). Expression of Flag-pVHL was confirmed by western blotting. Values are mean ± S.D. (*n* = 3). One-way ANOVA analysis with Dunnett's multiple comparisons test, **p < 0.01; ***p < 0.001; ****p < 0.0001. (**C**) TOPFlash luciferase assays in *VHL*-knockout HEK293T cells. pVHL protein levels were confirmed by western blotting. Values are mean ± S.D. (*n* = 3). Unpaired *t*-test, *p < 0.05; **p < 0.01; ***p < 0.001. (**D**) TOPFlash luciferase assays in BIO-treated *VHL*-knockout HEK293T cells. TOPFlash plasmid was cotransfected with *Renilla* plasmid into control or *VHL*-knockout cells. Wnt/β-catenin activity was induced by BIO (1 μM) for 4 h. Values are mean ± S.D. (*n* = 3). Unpaired *t*-test, *p < 0.05; **p < 0.01; ***p < 0.001; ****p < 0.0001. (**E**) The transcriptional levels of Wnt target gene *AXIN2*, *NKD1*, and *CCND1* in *VHL*-knockout HEK293T cells were analyzed by qRT-PCR. Values are mean ± S.D. (*n* = 3). Unpaired *t*-test, ns, not significant; *p < 0.05; **p < 0.01; ***p < 0.001. (**F**) The transcriptional levels of Wnt target gene *AXIN2* and *NKD1* in pVHL-overexpressing 786-O cells were analyzed by qRT-PCR. Values are mean ± S.D. (*n* = 3). Unpaired *t*-test, ns, not significant; *p < 0.05; **p < 0.01. (**G**) Endogenous TCF protein levels in HEK293T cells with increasing pVHL overexpression. Western blot analysis detected two distinct TCF7L2 isoforms: TCF7L2E and TCF7L2M/S. (H) Reintroduction of Flag-pVHL downregulated TCF7 and TCF7L2 in 786-O cells. (I) Flag-pVHL promotes endogenous TCF7L2 degradation in HEK293T cells. HEK293T cells were transfected with empty vector or Flag-pVHL, after 48 h, treated with cycloheximide (CHX; 100 μg mL.^−1^), and harvested at indicated time points (0, 2, 4, 8 h). Quantification of the total protein levels of the two TCF7L2 isoforms, TCF7L2E and TCF7L2M/S were normalized to Histone H3 (right panel). Values are mean ± S.D. (*n* = 3). Two-way ANOVA analysis with Bonferroni's multiple comparisons test, ns, not significant; *p < 0.05; ***p < 0.001. (J) The protein levels of TCF/LEF in control and *VHL*-Knockout cells. The expression level of HIF-1α was used as a positive control. Relative protein level normalized to Histone H3 (lower panel). Values are mean ± S.D. (*n* = 3). Unpaired *t*-test, *p < 0.05; **p < 0.01; ***p < 0.001. (K) The transcriptional levels of *TCF/LEF* in control and *VHL*-knockout cells were analyzed by qRT-PCR. Values are mean ± S.D. (*n* = 3). Unpaired *t*-test, ns, not significant. (L) Introduction of Flag-pVHL into *VHL*-knockout HEK293T cells downregulated TCF/LEF protein levels. Relative protein level normalized to Histone H3 (lower panel). SE, short time of exposure; LE, long time of exposure. Values are mean ± S.D. (*n* = 3). Unpaired *t*-test, *p < 0.05; **p < 0.01; ***p < 0.001

Our previous study reported that VBP1 regulates TCF/LEF stability through a pVHL-dependent mechanism [19]. To elucidate the underlying mechanism by which pVHL regulates TCF/LEF protein stability, we investigated the effects of pVHL on Wnt-induced TCF/LEF-dependent transcriptional activity. The HCT116 colorectal carcinoma cell line harbors a stabilized mutation in β-catenin that constitutively activates the Wnt/β-catenin signaling pathway [28]. A TOPFlash reporter plasmid, which contained Wnt-responsive TCF/LEF binding sites, was transfected into HCT116 cells, and then the transcriptional activity was measured. We observed that pVHL decreased expression of the TOPFlash reporter in HCT116 cells in a dose-dependent manner (Fig. 1A). These findings suggest that pVHL regulates Wnt signaling downstream of β-catenin. We next investigated whether pVHL modulates Wnt/β-catenin signaling at the TCF/LEF level. To this end, we utilized HEK293T cells, which possess a functional Wnt signaling system with basal Wnt/β-catenin signaling activity (referred to as"Wnt-off"state) [28], were co-transfected with pVHL and VP16-Tcf7l1ΔN, a constitutively active fusion protein derived from Tcf7l1 that lacks the β-catenin-binding domain and is therefore β-catenin-independent. Subsequently, Wnt reporter activity was evaluated. In agreement with the inhibitory effect of VBP1 on VP16-Tcf7l1ΔN-induced Wnt reporter activity previously reported [19], pVHL also inhibited VP16-Tcf7l1ΔN-induced Wnt reporter activity in a dose-dependent manner (Fig. 1B). These results suggested that pVHL inhibits Wnt reporter activity at the TCF/LEF level.

To further test the effect of pVHL on TCF/LEF, *VHL*-knockout HEK293T cells were generated using a CRISPR/Cas9-mediated gene editing approach (Fig. S1A and B). Two pVHL protein isoforms, a long form and a short form, have been previously reported [29]. The *VHL*-knockout cell lines were established, harboring either premature termination codons in exon 1 (lines #1–4) or a large deletion (line #5). Both types of mutations result in the depletion of both isoforms of VHL (Fig. 1C). Knockout of pVHL enhanced basal Wnt reporter activity (Fig. 1C). The addition of a GSK3 inhibitor, 6-bromoindirubin-3’-oxime (BIO), in HEK293T cells could induce Wnt reporter activity. Under BIO treatment, knockout of pVHL further increased Wnt reporter activity albeit with differently response degrees (Fig. 1D). To confirm this result, we further evaluated the transcriptional levels of Wnt target genes in several pVHL-depleted cell clones that exhibited relatively lower Wnt reporter activity responsiveness under BIO treatment. Indeed, the knockout of pVHL resulted in a significant increase in the mRNA levels of Wnt target genes, including *AXIN2*, *NKD1*, and *CCND1*, in these clones (Fig. 1E). This result further indicated that knockout of pVHL enhanced Wnt/β-catenin signaling activity. In addition, most cases of ccRCCs are associated with the inactivation of *VHL* [30], we therefore assessed the response of the *VHL*-deficient 786-O cell line to BIO treatment. BIO treatment significantly increased the mRNA expression levels of *AXIN2* and *NKD1* in 786-O cells; however, the reintroduction of pVHL attenuated this effect (Fig. 1F). Taken together, these results suggested that pVHL inhibits Wnt/β-catenin signaling.

We speculate that pVHL may promote VP16-Tcf7l1ΔN protein degradation and thus prevent its ability to induce the Wnt reporter activity. To test this hypothesis, we co-transfected Flag-tagged pVHL with Myc-tagged Tcf7, Tcf7l1, Tcf7l2, and Lef1 into HEK293T or HCT116 cells, respectively. The overexpression of pVHL reduced the abundance of Tcf7, Tcf7l1, Tcf7l2, and Lef1 in both cell lines (Fig. S2A and B). Additionally, pVHL also decreased the endogenous protein levels of TCF/LEF family members, including TCF7, TCF7L1, and both major TCF7L2 isoforms (TCF7L2E and TCF7L2M/S) in the HEK293T cells in a dose-dependent manner (Fig. 1G). Likewise, the reintroduction of pVHL into naturally *VHL*-deficient 786-O cells led to a reduction in the endogenous protein levels of TCF7 and TCF7L2 (Fig. 1H). Therefore, pVHL suppresses Wnt/β-catenin signaling by downregulating TCF/LEF proteins, indicating that this mechanism may play a role in the pathogenesis of ccRCC following the loss of pVHL. Taken together, these results suggested that pVHL negatively regulates Wnt/β-catenin activity and the TCF/LEF protein level.

To further investigate whether pVHL promotes the degradation of TCF/LEF proteins, we conducted a time-course treatment assay using cycloheximide (CHX), a protein synthesis inhibitor. When Myc-tagged pVHL was transfected, the degradation of endogenous TCF7L2 was significantly accelerated (Fig. 1I). In contrast, knockout of pVHL significantly increased not only HIF-1α, but also TCF7, TCF7L1, and TCF7L2 protein levels (Fig. 1J). However, knockout of pVHL did not alter the mRNA levels of *TCF7*, *TCF7L1*, *TCF7L2*, or *HIF-1α* (Fig. 1K). Moreover, reintroduction of pVHL into *VHL*-knockout HEK293T cells neutralized this effect, as the TCF7, TCF7L1, TCF7L2, and HIF-1α protein levels were reduced (Fig. 1L). These results indicate that the *VHL*-knockout is specific and that pVHL promotes the degradation of TCF/LEF proteins.

### pVHL interacts with TCF/LEF

**Fig. 2:**
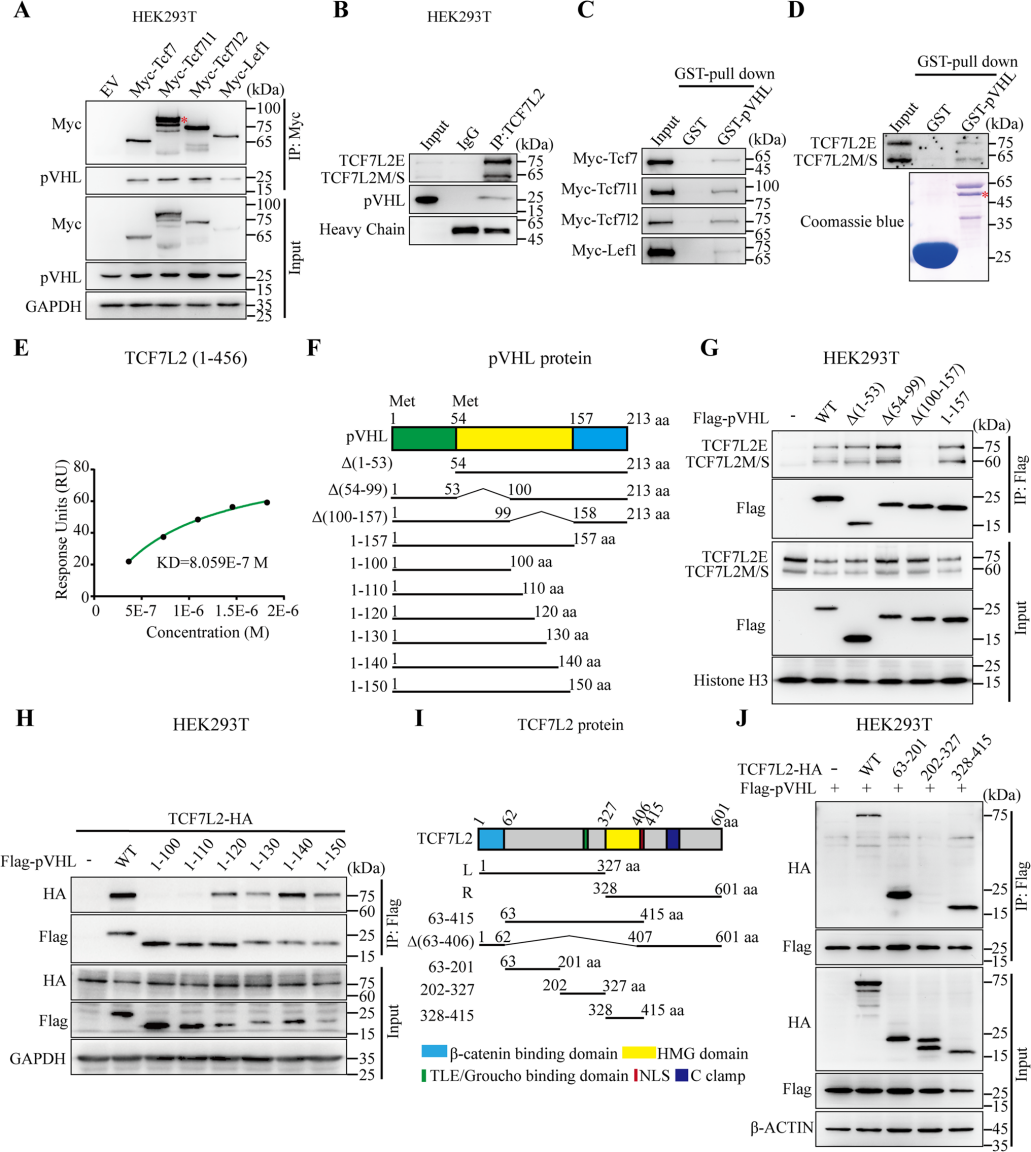
pVHL directly binds with TCF/LEF (**A**) Detection of pVHL binding to TCF/LEF in HEK293T cells by Co-IP. Red asterisk indicates the specific band. (**B**) Co-IP assay revealed the endogenous interaction between TCF7L2 and pVHL in HEK293T cells. (**C**, **D**) pVHL pulls down TCF/LEF. Purified GST or GST-pVHL proteins were incubated with extracts of HEK293T cells either transfected with Myc-Tcf/Lef (**C**) or untransfected (**D**). Bound proteins were eluted and analyzed by western blot using indicated antibodies. Red asterisk indicates GST-pVHL. (**E**) Surface plasmon resonance analysis of interactions between pVHL with TCF7L2(1–456) using purified recombinant proteins. (**F**) Schematic representations of pVHL and truncated mutant proteins. (**G** and **H**) Mapping pVHL binding domain interacting with endogenous or exogenous TCF7L2 in transfected HEK293T cells by Co-IP assay. (**I**) Schematic representation of of TCF7L2 WT and truncated mutant proteins. (**J**) Mapping TCF7L2 binding domain interacting with pVHL in transfected HEK293T cells by Co-IP assay

We next examined whether TCF/LEF and pVHL interact with each other. Co-IP assay showed that endogenous pVHL interacts with all four TCF/LEF members (Fig. 2A). In addition, co-IP assay also indicated that endogenous TCF7L2 retrieved endogenous pVHL in HEK293T cells (Fig. 2B). Furthermore, purified glutathione-S-transferase (GST)-pVHL protein pulled down all four members of Myc-tagged TCF/LEF in vitro (Fig. 2C). Likewise, a protein–protein interaction between pVHL and endogenous TCF7L2 was also confirmed (Fig. 2D). To further confirm the interaction between pVHL and TCF7L2, surface plasmon resonance analysis was performed using purified recombinant pVHL and TCF7L2(1–456) proteins. As shown in Fig. 2E, pVHL directly binds to TCF7L2(1–456). Collectively, these data revealed that TCF/LEF and pVHL directly interact with each other.

To identify the pVHL domain(s) essential for TCF/LEF interaction, various pVHL domain-deleted mutants were generated (Fig. 2F). Using Co-IP assay, we mapped the domain(s) putatively responsible for the interaction between pVHL and TCF7L2. A region comprising amino acid (aa) residues 100–157 was required for its interaction with endogenous TCF7L2 (Fig. 2G). Moreover, structural analysis revealed that the aa (111–120) is essential for pVHL-TCF7L2 binding, as pVHL (1–120), but not pVHL (1–110), was capable of co-immunoprecipitating TCF7L2 (Fig. 2H).

We next mapped the binding domain(s) of TCFL2 to pVHL. A variety of mutants of TCF7L2 were generated based on conserved functional motifs (Fig. 2I and S3). To ensure the nuclear localization of each deletion mutant of TCF7L2, all of the mutants contained an NLS. Co-IP analysis showed that aa 63–201 or aa 328–415, rather than other regions of TCF7L2, binds to pVHL (Fig. 2J and Fig. S4A, B). Immunostaining analysis confirmed that the interactions are specific, as the mutants TCF7L2 (63–201), TCF7L2 (202–327), and TCF7L2 (328–415) are all localized in the nucleus (Fig. S4C). These results suggested that aa 63–201 or aa 328–415 of TCF7L2 is required for interaction with pVHL. The aa 328–415 of TCF7L2 constitutes the HMG DNA-binding domain (HMG DBD), which is composed of HMG domain and NLS. The HMG DBD of TCF/LEF is evolutionarily conserved and nearly identical from invertebrate to vertebrate (Fig. S3) [9]. We therefore investigated whether HMG DBD was downregulated by pVHL. As expected, the Tcf7l1-HMG DBD level was reduced by pVHL overexpression (Fig. S4D). Given that the HMG domain recognizes and binds to specific DNA sequences, we investigated whether pVHL inhibits the binding of TCF/LEF proteins to DNA. Indeed, the ChIP-qPCR analysis revealed that the knockout of pVHL had minimal impact on the binding of TCF7L2 to the target promoter regions of *AXIN2* and *NKD1* (Fig. S4E). Collectively, our results indicate that aa 63–201 or 328–415 of TCF7L2 is crucial for pVHL binding.

### pVHL promotes the proteasomal degradation of TCF/LEF through a mechanism independent of the ubiquitin-mediated pathway

As mentioned earlier, pVHL recognizes and binds to prolyl-hydroxylated substrates, such as prolyl-hydroxylated HIF-α, Akt, and ZHX2, in order to exert its function. Three residues (S111, H115, and W117) in the pVHL hydroxyl-proline binding pocket are critical for pVHL interaction with prolyl-hydroxylated substrates [14]. This triple residue-mutated pVHL was utilized to test whether it has comparable functionality with that of the WT pVHL. Like WT pVHL, the pVHL mutant also downregulated the abundance of Tcf7L2 (Fig. S5A). We next applied a prolyl hydroxylase inhibitor, DMOG, to inhibit the activity of EGLN 1/2/3. It has been reported that DMOG treatment inhibits the binding between HIF-2α and pVHL and therefore stabilizes HIF-2α [14]. However, DMOG treatment did not reverse TCF7L2 protein downregulation induced by pVHL overexpression (Fig. S5B). Therefore, TCF7L2 degradation by pVHL does not depend on prolyl hydroxylation of TCF7L2.

A previous report has indicated that chronic starvation-stimulated autophagy negatively regulates Wnt/β-catenin signaling [31]. We examined the effects of starvation, an autophagy stimulus with nutrient deprivation medium, on the expression of endogenous TCF7L2 in HEK293T cells. Chronic starvation reduced the protein levels of both non-p-β-catenin and total β-catenin but not that of TCF7L2 (Fig. S5C). This result suggested that TCF7L2 is not degraded by autophagy.

To exclude the effect of increased HIFs on TCF7, TCF7L1, and TCF7L2 in *VHL*-knockout HEK293T cells, we examined the protein levels of TCF7, TCF7L1, and TCF7L2 with enhanced HIF-1α expression upon hypoxia treatment. Hypoxia treatment increased the protein level of HIF-1α, while the protein levels of TCF7, TCF7L1, and TCF7L2 were not increased (Fig. S6A). Dimerization of HIF-1α or HIF-2α with HIF-1β is mediated by their basic helix-loop-helix (bHLH) and PER-ARNT-SIM (PAS) domains, which are required for binding to hypoxia response elements (HREs) and HIF-dependent transcriptional activity [32]. In this case, we generated *HIF-1β* (*ARNT*) knockout HEK293T cells, targeting exon 6 to disrupt its bHLH and PAS domains (Fig. S6B and C). Indeed, the cells with absence of HIF-1β did not increase protein levels of TCF7, TCF7L1, and TCF7L2 under hypoxia treatment (Fig. S6D). Therefore, the HIF activity did not upregulate the protein levels of TCF7, TCF7L1, and TCF7L2.

**Fig. 3:**
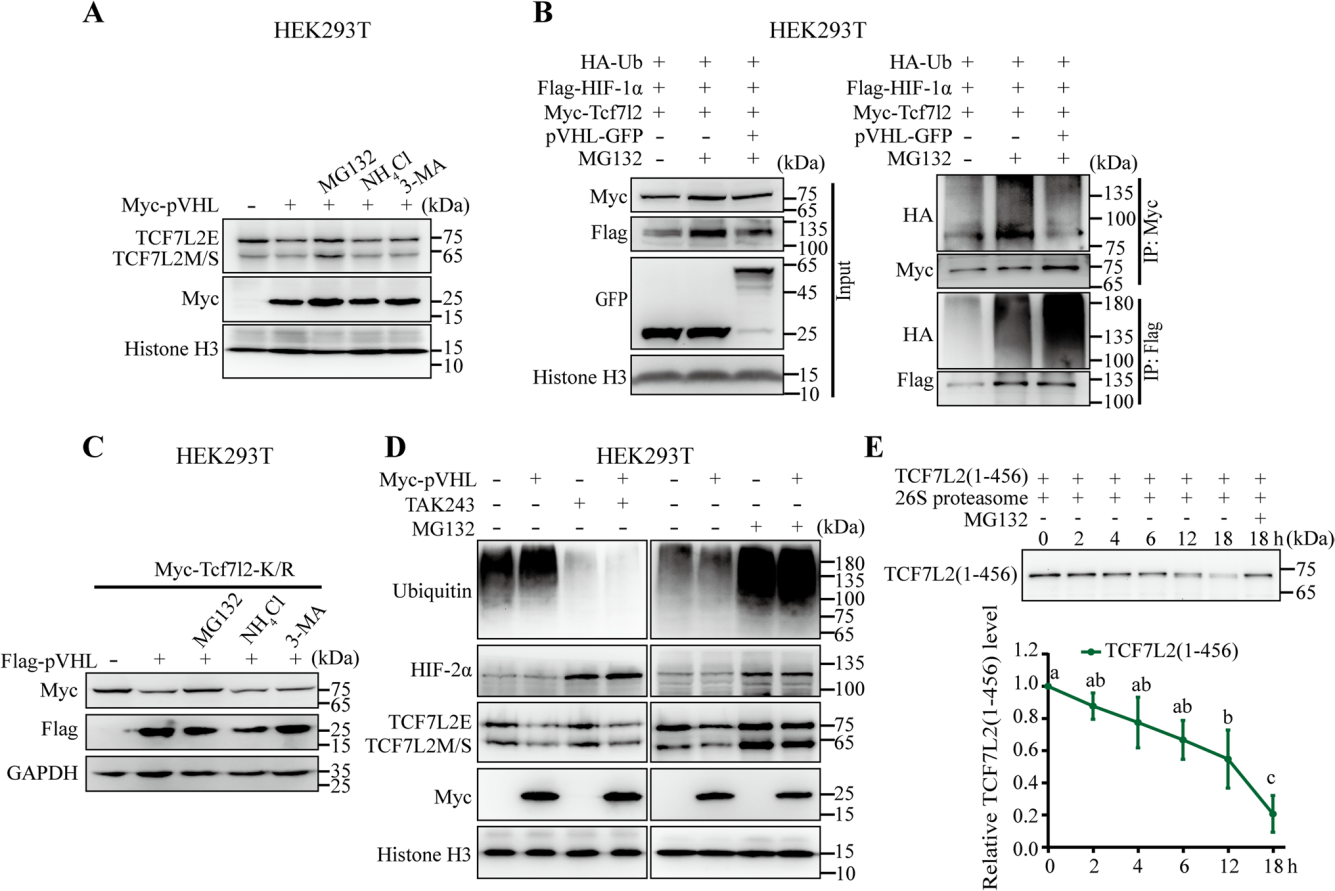
pVHL promotes TCF/LEF degradation by ubiquitin-independent proteasome pathway (**A**) Changes in endogenous TCF7L2 protein levels in pVHL-overexpressing HEK293T cells treated with indicated inhibitors. The transfected cells were either untreated or treated with MG132 (10 μM), NH_4_Cl (25 mM), or 3-MA (5 mM) for 8 h. (**B**) Effects of pVHL-overexpression on Tcf7l2 ubiquitination. Myc-Tcf7l2, Flag-HIF-1α, and HA-Ub were co-transfected with GFP-Vector or pVHL-GFP into HEK293T cells. After 48 h, cells were treated with MG132 (10 μM) for 8 h, and lysed for immunoprecipitation with anti-Myc and anti-Flag antibody. (**C**) Changes in Tcf7l2-K/R protein levels in pVHL-overexpressing HEK293T cells treated with indicated inhibitors. Western blot analysis of whole cell lysis derived from HEK293T cells transfected with indicated plasmid DNA and either untreated or treated with MG132 (10 μM), NH_4_Cl (25 mM), or 3-MA (5 mM) for 8 h. (**D**) Changes in endogenous TCF7L2 or HIF-2α protein levels in pVHL-overexpressing HEK293T cells treated with a specific inhibitor for the ubiquitin activating enzyme or proteasome. The transfected cells were either untreated or treated with TAK243 (1 μM) for 12 h or MG132 (10 μM) for 8 h. (**E**) Degradation analysis of TCF7L2(1–456) protein in cell-free system. The amount of TCF7L2(1–456) protein degraded by 26S proteasome at each indicated time points (0, 2, 4, 6, 12, 18 h) in cell-free system. The group with MG132 treatment was used as a control. Values are mean ± S.D. (*n* = 3). One-way ANOVA analysis with Tukey's multiple comparisons test. Groups marked with distinct letters show significant differences from one another (*p* < 0.05)

To address the possible pathway of TCF/LEF degradation, we used specific small compound inhibitors, including MG132 (proteasomal inhibitor), NH_4_Cl (lysosomal proteolysis inhibitor), and 3-MA (autophagy inhibitor), to block the major protein degradation pathway. Addition of MG132 but not of NH_4_Cl or 3-MA blocked pVHL-mediated TCF7L2 degradation (Fig. 3A). Thus, it is likely that pVHL promotes TCF7L2 degradation via the proteasomal pathway.

Given that pVHL functions as an E3 ubiquitin ligase, we subsequently investigated the effects of pVHL on the ubiquitylation of TCF7L2. Overexpression of pVHL enhanced the polyubiquitination level of HIF-1α but not Tcf7l2 (Fig. 3B). Therefore, during the regulation of TCF7L2 protein stability, pVHL does not exhibit canonical functionality as a conventional E3 ubiquitin ligase, which typically catalyzes the formation of polyubiquitin chains on target proteins. Instead, pVHL likely downregulates TCF7L2 through a mechanism distinct from its regulation of HIF-1α, suggesting a unique mode of action.

We further analyzed evolutionarily conserved lysine residues in several vertebrate TCF/LEF proteins and found approximately 19 conserved lysine residues in total (Fig. S3). We mutated all of them into arginine residues (R) in a Myc-tagged *Xenopus* Tcf7l2 background (hereafter, Tcf7l2-K/R). Indeed, pVHL also reduced the protein level of the Tcf7l2-K/R mutant. Moreover, addition of MG132 but not of NH_4_Cl or 3-MA blocked pVHL-mediated Tcf7l2-K/R mutant degradation (Fig. 3C). Collectively, these results suggested that pVHL likely promotes TCF7L2 proteasomal degradation independently of classical lysine-dependent ubiquitin function.

In addition to the classical lysine-dependent ubiquitin modification, lysine-independent N-terminal ubiquitin modification or serine, threonine, cysteine, and tyrosine-dependent ubiquitin modification is also involved in a variety of protein stability regulation. All of the above ubiquitin modifications require ubiquitin activation [33, 34]. To exclude the above non-classical lysine-independent ubiquitin modification, we tested whether reduction of TCF7L2 protein levels under pVHL overexpression were restored by inhibiting activation of ubiquitin. Indeed, addition of TAK-243, a ubiquitin activating enzyme (UAE)-specific inhibitor, restored protein levels of HIF-2α while not TCF7L2 (Fig. 3D). In contrast, addition of proteasomal inhibitor MG132 restored protein levels of both in pVHL-overexpressing cells (Fig. 3D). The results indicate that pVHL likely promotes TCF7L2 degradation in a proteasome-dependent but ubiquitin activation-independent manner.

Some proteins, such as retinoblastoma protein and HIF, have been reported to be degraded through the ubiquitin-independent proteasome pathway. These proteins can be directly degraded when incubated with purified proteasomes in cell-free systems [18, 35]. To further confirm that TCF7L2 undergoes ubiquitin-independent proteasomal degradation, we investigated whether purified TCF7L2 protein with any modifications could be directly degraded by the 26S proteasome in a cell-free system. To this end, the recombinant TCF7L2(1–456) protein was incubated with the purified 26S proteasome in a cell-free system. We assessed the degradation rate of recombinant TCF7L2(1–456) protein in the presence of purified 26S proteasome. The abundance of TCF7L2(1–456) decreased gradually in a time-dependent manner, indicating that TCF7L2(1–456) was directly degraded by the 26S proteasome in the cell-free system (Fig. 3E). Collectively, these findings suggest that the downregulatory effect of pVHL on TCF/LEF is independent of ubiquitin-mediated mechanisms.

### The pVHL downregulates the TCF/LEF proteins through a VBC complex-independent pathway

**Fig. 4:**
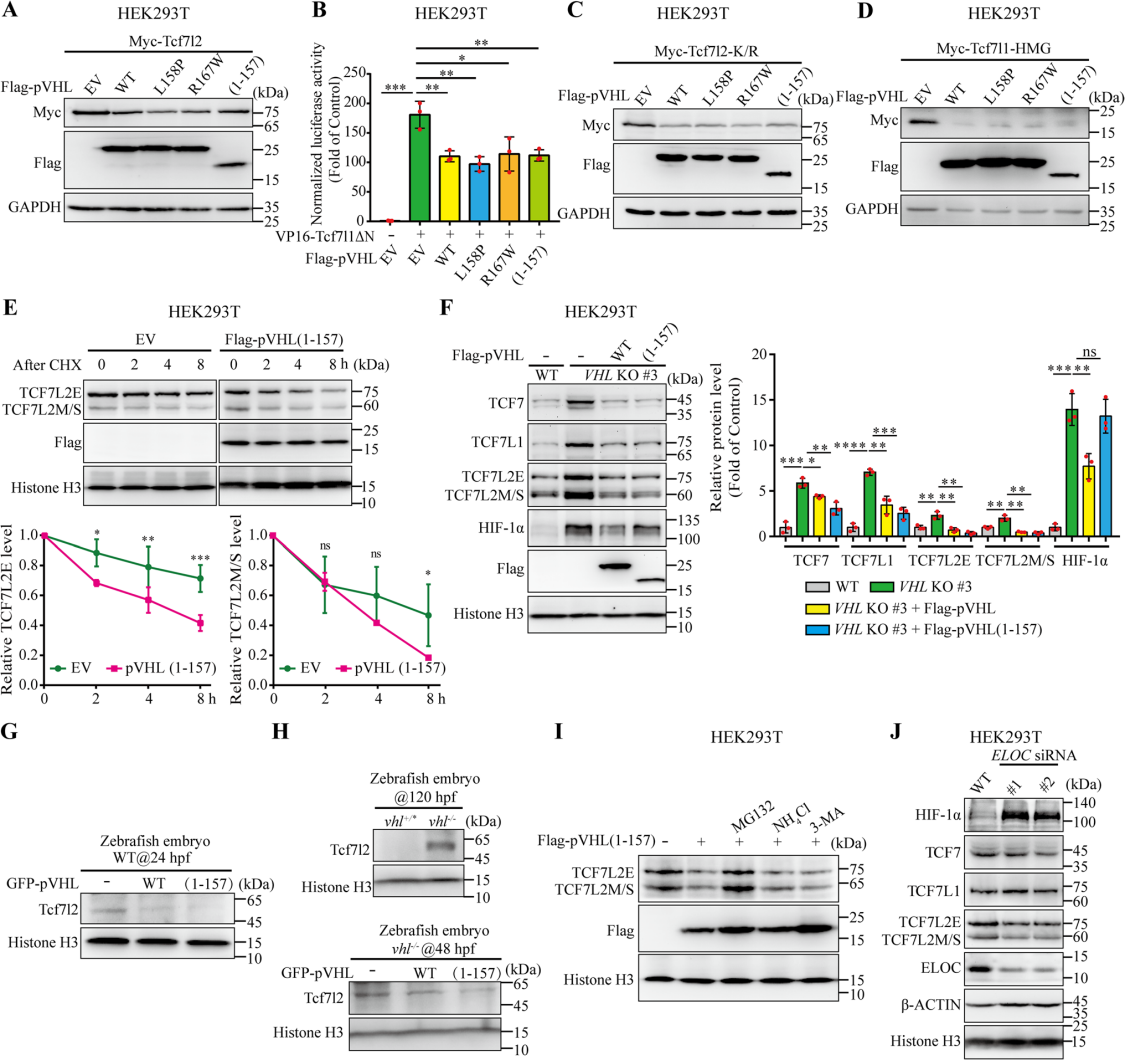
pVHL promotes TCF degradation in an E3 ubiquitin ligase-independent manner (**A**) Tcf7l2 protein levels in HEK293T cells with overexpression of WT, site-mutated, or truncated pVHL. (**B**) TOPFlash reporter assays in VP16-Tcf7l1ΔN-transfected HEK293T cells with overexpression of WT, site-mutated, or truncated pVHL. Wnt/β-catenin signal was activated by transfection with VP16-Tcf7l1ΔN (50 ng). Values are mean ± S.D. (*n* = 3). Unpaired *t*-test. *p < 0.05; **p < 0.01; ***p < 0.001. (**C**) Tcf7l2-K/R protein levels in HEK293T cells with overexpression of WT, site-mutated, or truncated pVHL. (**D**) Tcf7l1-HMG DBD protein levels in HEK293T cells with overexpression of WT, site-mutated, or truncated pVHL. (**E**) pVHL truncation mutant pVHL (1–157) promotes endogenous TCF7L2 degradation in HEK293T cells. HEK293T cells were transfected with empty vector or Flag-pVHL (1–157), after 48 h, treated with cycloheximide (CHX; 100 μg mL^−1^) and harvested at indicated time points (0, 2, 4, 8 h). The total protein levels of the two TCF7L2 isoforms, TCF7L2E and TCF7L2M/S, were normalized to Histone H3 (lower panel). Values are mean ± S.D. (*n* = 3). Two-way ANOVA analysis with Bonferroni's multiple comparisons test. ns, not significant; *p < 0.05; **p < 0.01; ****p < 0.0001. (**F**) Overexpression of Flag-pVHL and Flag-pVHL (1–157) reduced TCF7, TCF7L1, and TCF7L2 protein levels in *VHL*-KO cells. HIF-1α was downregulated in *VHL*-KO after transfection with Flag-pVHL but not with Flag-pVHL (1–157). Relative protein level normalized to Histone H3 (right panel). Values are mean ± S.D. (*n* = 3). Unpaired *t*-test, ns, not significant; *p < 0.05; **p < 0.01; ***p < 0.001. (**G**) Overexpression of pVHL or pVHL (1–157) decreased endogenous Tcf7l2 protein level in wide-type zebrafish embryos at 24 hpf. Protein samples of 4 zebrafish embryos were added in each well. (**H**) *vhl*-null mutant zebrafish embryos exhibited elevated protein levels of Tcf7l2 at 120 hpf (upper panel). Reintroduction of pVHL or pVHL (1–157) into *vhl*-null mutant zebrafish embryos reduced Tcf7l2 protein level at 48 hpf (lower panel). Protein samples of 4 zebrafish embryos were added in each well. (**I**) Changes in endogenous TCF7L2 protein levels in pVHL (1–157)-overexpressing HEK293T cells treated with indicated inhibitors. The transfected cells were either untreated or treated with MG132 (10 μM), NH_4_Cl (25 mM), or 3-MA (5 mM) for 8 h. (**J**) The protein levels of TCF/LEF in control or *ELOC*-Knockdown cells. The expression level of HIF-1α was used as a positive control

To further validate that pVHL-mediated TCF/LEF degradation does not rely on E3 ubiquitin ligase activity, we tested the effects on TCF/LEF by naturally occurring and cancer-associated pVHL point mutants L158P and R167W and the truncated mutant pVHL (1–157). All three mutants reduced or diminished elongin B/C binding capability and abolished E3 ligase activity [36–38]. They all exhibited the same effects on the abundance of Tcf7l2 protein and VP16-Tcf7l1ΔN-induced Wnt reporter activity as WT pVHL (Fig. 4A and B). Likewise, they all reduced the Tcf7l2-K/R mutant protein levels (Fig. 4C). Similar effects were observed when each mutant was co-expressed with Tcf7l1-HMG DBD (Fig. 4D).

We next test the effects of pVHL (1–157) on protein levels of TCF/LEF at endogenous level. As WT pVHL, pVHL (1–157) also significantly downregulated TCF7L2 protein and shortened its half-life (Fig. 4E). Likewise, introduction of pVHL (1–157) into *VHL*-knockout HEK293T cells remarkably decreased TCF7, TCF7L1, and TCF7L2 protein accumulation by depleting pVHL, while the HIF-1α protein level was reduced by WT pVHL rather than by the pVHL (1–157) mutant (Fig. 4F). Therefore, pVHL (1–157) and WT pVHL had comparable effects on TCF/LEF downregulation. In addition, we used developing zebrafish embryos to determine the effects of human pVHL and pVHL (1–157) on the promotion of Tcf7l2 degradation *in vivo*. We generated *in vitro* transcribed *GFP*, *VHL-P2A-GFP*, or *VHL (1*–*157)-P2A-GFP* mRNA and injected them into zebrafish embryos. The Tcf7l2 protein levels were reduced in the zebrafish embryos injected with either *VHL-P2A-GFP* or *VHL (1*–*157)-P2A-GFP* mRNA (Fig. 4G). Zebrafish pVhl is an ortholog of the short human pVHL isoform [39]. Knockout of pVhl in zebrafish caused accumulation of Tcf7l2 at the larval stage (120 hpf) (Fig. 4H, upper panel). Introduction of human *VHL* mRNA also decreased Tcf7l2 protein levels in *vhl*-null mutant background at 48 hpf (Fig. 4H, lower panel). The pVHL (1–157) mRNA had a similar effect (Fig. 4H, lower panel). Additionally, the endogenous protein levels of TCF7L2 were also reduced by pVHL (1–157), and this reduction was blocked by addition of MG132, but not of NH_4_Cl or 3-MA (Fig. 4I). Taken together, these results suggested that pVHL (1–157) is sufficient to reduced protein levels of TCF/LEF via proteasomal degradation.

We next test this possibility at endogenous level. As mentioned above, pVHL functions as a subunit of an E3 ligase complex to recognize substrates. In this complex, pVHL, Elongin B/C, and RBX1 in association with ubiquitin-conjugated E2 component, are assembled by CUL2 to ubiquitinate pVHL-bound HIF-α proteins [40]. Depletion of Elongin C leads to disassembly of the E3 ligase complex, which resulted in accumulation of HIF-α proteins [41]. We tested whether the proteasomal degradation of TCF/LEF depend on the whole VBC complex as HIF-α proteins. In agreement with previous results, *ELOC*-knockdown increased HIF-1α proteins; however, the protein levels of TCF7, TCF7L1, and TCF7L2 did not accumulate simultaneously (Fig. 4J). These results implied that the whole VBC complex is not necessary for the maintenance of TCF/LEF protein stability.

The above results prompted us to hypothesize whether any naturally occurring isoforms or homologues of pVHL exist that abolish E3 ligase activity. We noted that a previous study reported the existence of a human pVHL homologue, the pVHL-like protein (pVHLL; also known as pVLP), which lacks the domain responsible for Elongin C binding (corresponding to aa 1–160 in pVHL) (Fig. S7A, B) [42]. Unlike pVHL, pVHLL has an incapability for assembling an E3 ubiquitin ligase complex that consists of Elongin B/C, RBX1, and CUL2. Consequently, pVHLL acts as a dominant-negative pVHL to promote the accumulation of HIF-1α since it binds to HIF-1α independent of prolyl hydroxylation status [42]. We speculate that pVHLL exerts a similar effect on the protein stability of TCF/LEF as both the WT and mutated forms of pVHL. Indeed, endogenous TCF7L2 is associated with Flag-tagged pVHLL in HEK293T cells (Fig. S7C). As expected, the endogenous protein levels of TCF7, TCF7L1, and TCF7L2 were reduced by pVHLL in HEK293T cells (Fig. S7D). These results suggested that pVHLL reduce protein levels of TCF/LEF. Thus, substrate recognition by pVHL as a component of E3 ubiquitin ligase complex is not required for TCF/LEF protein degradation.

Collectively, these data implied that pVHL does not function as an E3 ligase complex adaptor in VBC complex to promote TCF degradation.

### pVHL directly interacts with the 26S proteasome to mediate TCF7L2 degradation

**Fig. 5:**
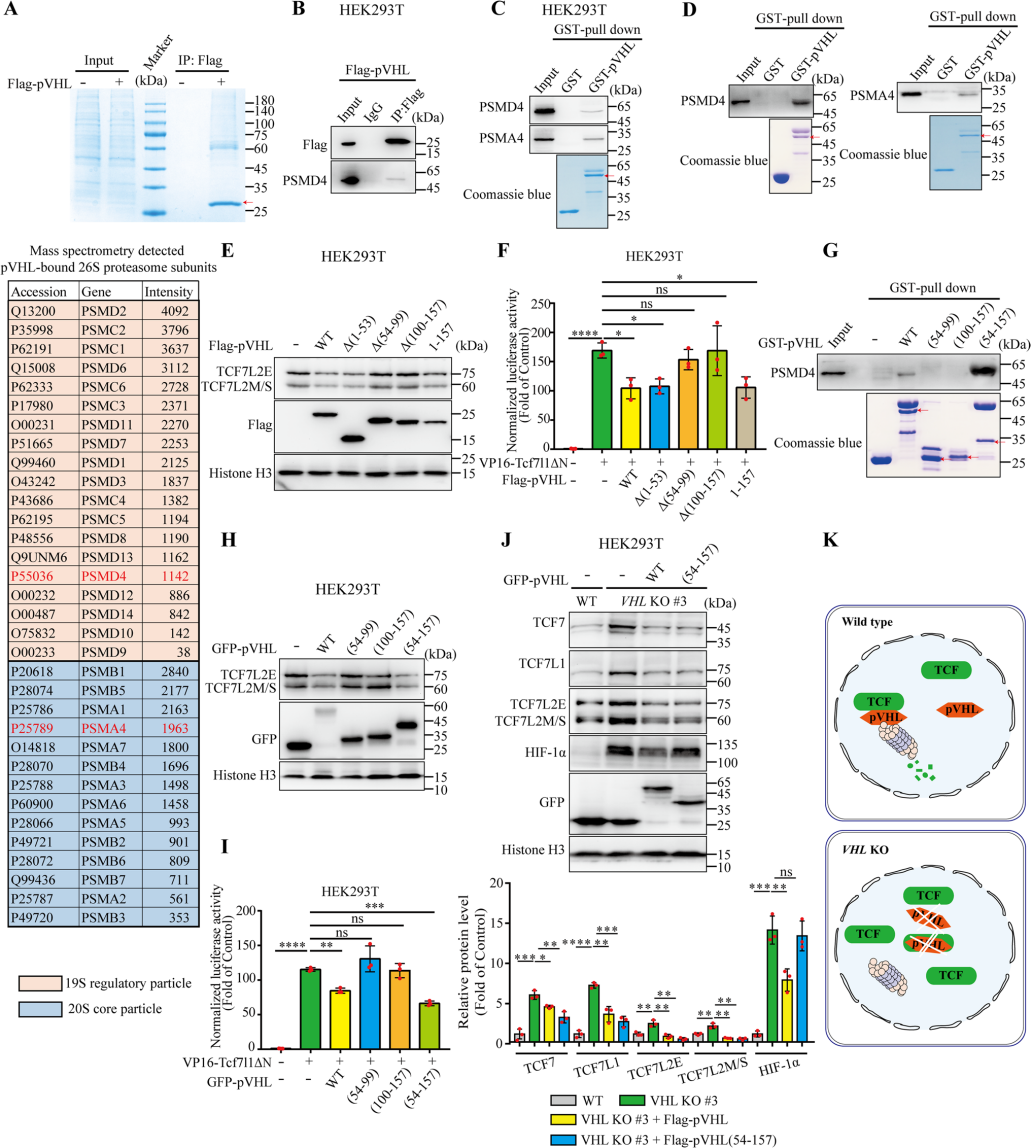
pVHL directly interacts with 26S proteasome to promote TCF protein degradation (**A**) IP-MS analyzing the proteins interacting with pVHL in HEK293T cells. After transfecting pCS2-Flag or pCS2-Flag-pVHL plasmids into HEK293T cells for 48 h, cells were treated with MG132 (10 μM) for 8 h. Flag-pVHL was then immunoprecipitated using anti-Flag antibody and analyzed by mass spectrometry. Coomassie blue staining of Flag immunoprecipitates revealed proteins interacting with Flag-pVHL. Red arrow indicates Flag-pVHL. Mass spectrometry analysis identified multiple subunits of the 26S proteasome, including PSMD4 (19S regulatory particle) and PSMA4 (20S core particle), which are highlighted in red in the table (lower panel). (**B**) Co-IP assay revealed that Flag-pVHL interacted with 26S proteasome, as indicated by detecting 19S regulatory subunit PSMD4 in HEK293T cells with an antibody against PSMD4. (**C**) pVHL pulls down 26S proteasome, as indicated by detecting 19S regulatory subunit PSMD4 and 20S core particle PSMA4 in HEK293T cells. Purified GST or GST-pVHL protein was incubated with extracts of HEK293T cells. Red arrow indicates GST-pVHL. (**D**) *In vitro* cell-free GST pulldown assay revealed direct interaction between pVHL and 26S proteasome. Purified GST or GST-pVHL was incubated with recombinant 26S proteasome. Red arrow indicates GST-pVHL. (**E**) Endogenous TCF7L2 protein levels in HEK293T cells with overexpression of indicated pVHL mutants. (**F**) TOPFlash reporter assays in HEK293T cells with coexpression of VP16-Tcf7l1ΔN and each indicated pVHL mutant. Wnt/β-catenin signal was activated by transfection with VP16-Tcf7l1ΔN (50 ng). Values are mean ± S.D. (*n* = 3). One-way ANOVA analysis with Dunnett's multiple comparisons test. ns, not significant; *p < 0.05; ****p < 0.0001. (**G**) *In vitro* cell-free GST pulldown assay detected binding of full-length and mutant pVHL to 26S proteasome. Red arrows indicate GST-fusion protein. (**H**) Endogenous TCF7L2 protein levels in HEK293T cells with overexpression of indicated pVHL mutants. (**I**) TOPFlash reporter assays in HEK293T cells with coexpression of VP16-Tcf7l1ΔN and each indicated pVHL mutant. Wnt/β-catenin signal was activated by transfection with VP16-Tcf7l1ΔN (50 ng). Values are mean ± S.D. (*n* = 3). One-way ANOVA analysis with Dunnett's multiple comparisons test. ns, not significant; ** *p* < 0.01; ***p < 0.001; ****p < 0.0001. (**J**) Introduction of pVHL and pVHL(54–157) into *VHL*-KO cells reduced TCF7, TCF7L1, and TCF7L2 protein levels. HIF-1α was downregulated in *VHL*-KO after transfection with pVHL-GFP but not with pVHL(54–157)-GFP. Relative protein level normalized to Histone H3 (lower panel). Values are mean ± S.D. (*n* = 3). Unpaired *t*-test, ns, not significant; *p < 0.05; **p < 0.01; ***p < 0.001; ****p < 0.0001. (**K**) Schematic illustration of the mechanism by which TCF/LEF protein stability is regulated by pVHL

The proteasome primarily functions in the degradation of ubiquitin-modified proteins and is also capable of processing non-ubiquitinated substrates [43]. Several proteins, including Parkin, Rad23, and midnolin, are likely to interact with the 26S proteasome and form complexes that mediate substrate degradation [44–47]. Given that pVHL (1–157) is sufficient to promote the proteasomal degradation of TCF/LEF and that purified, unmodified TCF7L2 protein is directly degraded by the purified 26S proteasome in a cell-free system, we hypothesized that pVHL mediates ubiquitin-independent proteasomal degradation by bridging TCF/LEF to the proteasome, thereby facilitating their degradation. To test this hypothesis, we conducted IP-MS analysis using HEK293T cells expressing Flag-pVHL to determine whether proteins of the 26S proteasome could be enriched by a Flag antibody. The 26S proteasome consists of the 19S regulatory particle and the 20S core particle and each particle contains multiple subunits [43]. As expected, 19 subunit proteins of the 19S regulatory particle and 14 subunit proteins of the 20S core particle were identified through IP-MS (Fig. 5A). Furthermore, we performed Co-IP and GST pull-down assays to validate the IP-MS results using representative proteins identified from the 19S regulatory particle and the 20S core particle, respectively. Co-IP assay showed that PSMD4 of 19S regulatory particle was retrieved by Flag-pVHL (Fig. 5B). In consistent, GST-pulldown assay with HEK293T cell lysis showed that recombinant GST-pVHL pulled down PSMD4 of 19S regulatory particle and PSMA4 of 20S core particle, respectively (Fig. 5C). Moreover, a cell-free system pulldown assay on purified human 26S proteasome and recombinant GST-pVHL also showed that pVHL pulled down PSMD4 or PSMA4 (Fig. 5D). Therefore, pVHL do directly interact with the 26S proteasome.

We subsequently mapped the pVHL domain(s) required for promoting TCF7L2 degradation. Deletion of aa 54–99 or 100–157 in pVHL attenuated its ability to downregulate endogenous TCF7L2 protein levels (Fig. 5E). Furthermore, deletion of either region in pVHL reduced its inhibitory effect on VP16-Tcf7l1ΔN-induced Wnt reporter activity (Fig. 5F). Therefore, aa 54–99 and 100–157 of pVHL are necessary for promoting TCF degradation. We subsequently investigated which region is vital for the interaction between pVHL and the 26S proteasome. The cell-free system pulldown assays showed that neither recombinant GST-pVHL (54–99) nor GST-pVHL (100–157) pulls down PSMD4 in purified human 26S proteasome (Fig. 5G). In contrast, GST-pVHL (54–157) indeed pulls down PSMD4, suggesting a direct interaction between aa 54–157 of pVHL and the 26S proteasome (Fig. 5G). Similarly, neither pVHL (54–99) nor pVHL (100–157) reduced endogenous TCF7L2 protein levels (Fig. 5H). However, the pVHL (54–157) fragment exhibited comparable efficacy to full-length pVHL in promoting TCF7L2 degradation (Fig. 5H). pVHL (54–157) but not pVHL (54–99) or pVHL (100–157) inhibited VP16-Tcf7l1ΔN-induced TOPFlash reporter activity (Fig. 5I). Additionally, pVHL (54–157) had the same capability as WT pVHL to significantly reduce protein levels of TCF7, TCF7L1, and TCF7L2 in pVHL-depleted HEK293T cells, while such effects were not observed on HIF-1α protein levels (Fig. 5J). These results implied that pVHL (54–157) is necessary and sufficient to promote TCF protein degradation. Taken together, these results suggested that the aa 54–157 of pVHL likely functions as a scaffold that recruits the TCF/LEF and 26S proteasome to mediate TCF degradation.

pVHL (54–157) is sufficient to mediate the degradation of TCF/LEF proteins. Purified TCF7L2 protein is directly degraded in the presence of the 26S proteasome in a cell-free system, and this degradation does not require ubiquitination. pVHL associates with the 26S proteasome and promotes the degradation of TCF/LEF proteins that are bound to it. Integrating the aforementioned cell-based and cell-free results, we conclude that pVHL likely functions as a bridge to facilitate the recruitment of TCF/LEF proteins to the proteasome for their degradation via a ubiquitin-independent mechanism (Fig. 5K).

### The pVHL (1–157) is sufficient to suppress cellular proliferation, and ventralize zebrafish embryos

We subsequently examined the cellular and in vivo consequences of the interactions between pVHL and TCF/LEF. Wnt/β-catenin signaling promotes cell proliferation. Considering that the activation of Wnt/β-catenin signaling enhances cell proliferation and that pVHL destabilizes TCF/LEF to suppress this pathway, we assessed the proliferative capacity of Wnt-activated HCT116 cells by modulating TCF/LEF protein levels through the alteration of pVHL expression. pVHL regulates multiple proliferation-related factors, including HIF, SMAD3, AKT, and ZHX2, through its canonical function within the VBC complex [14–17, 48]. Hence, it is challenging to exclude the potential influence of other pathways when employing a loss-of-function approach. We therefore evaluated the proliferative capacity of HCT116 cells by expressing pVHL (1–157) or pVHLL, neither of which can form a functional VBC complex, rather than expressing WT pVHL. Cells expressing pVHL (1–157) exhibited a significantly reduced proliferative capacity (Fig. S8). Similarly, a comparable effect was observed in HCT116 cells expressing pVHLL (Fig. S8). Collectively, these findings support the conclusion that pVHL suppresses cell proliferation, at least in part, through the destabilization of TCF/LEF transcription factors.

**Fig. 6:**
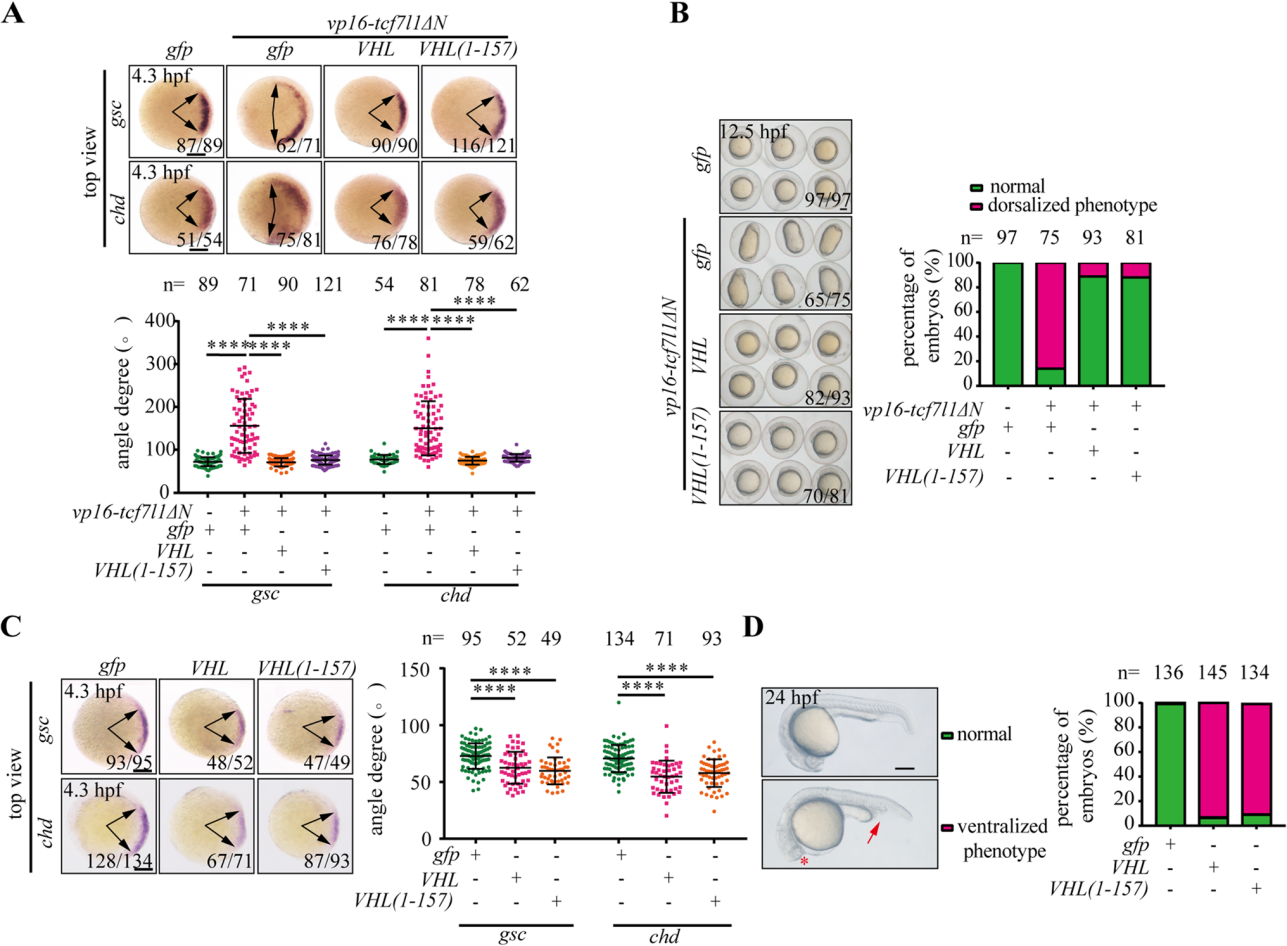
The pVHL (1–157) is sufficient to ventralize zebrafish embryos (**A**) Expression patterns of each indicated dorsal marker in *vp16-tcf7l1ΔN* mRNA- or *vp16-tcf7l1ΔN* mRNA plus *VHL* or *VHL (1–157)* mRNA-injected embryos at 4.3 hpf. The edges of specific markers are indicated by arrows. Top views with dorsal to the right. Quantification of the arc of marker expression shown below the representative embryo images (lower panel). The total number of embryos of each group are given at the top. Similar results were obtained from three experiments. Values are means ± S.D. ****p < 0.0001. Unpaired *t* test, two-tailed. Scale bar = 200 μm. (**B**) pVHL and pVHL (1–157) exhibit comparable antagonizing effect on VP16-Tcf7l1ΔN action *in vivo*. Representative images of embryos injected with of *gfp* (150 pg), the mRNA of of *vp16-tcf7l1ΔN* (50 pg), and mRNA of *vp16-tcf7l1ΔN* plus *VHL* or *VHL (1–157)* mRNA (100 pg) at 12.5 hpf. Quantitative results showing the representative embryo images (right panel). The results are from three independent experiments and the total embryo number is given at the top. Scale bar = 200 μm. (**C**) Expression patterns of each indicated dorsal marker in *gfp*, *VHL*, or *VHL (1–157)* mRNA-injected embryos at 4.3 hpf. The edges of specific markers are indicated by arrows. Top views with dorsal to the right. Quantification of the arc of marker expression showing the representative embryo images (right panel). The total number of embryos of each group are given at the top. Similar results were obtained from three experiments. Values are means ± S.D. ****p < 0.0001. Unpaired *t* test, two-tailed. Scale bar = 200 μm. (**D**) Representative images of embryos injected with *gfp*, *VHL*, or *VHL (1–157)* mRNA (500 pg) at 24 hpf. The reduced head tissue is indicated by asterisk and the enlarged ventral tissue is indicated by arrow. Quantitative results were presented in the right panel. The results are from three independent experiments and the total embryo number is given at the top. Scale bar = 200 μm

We next investigated the *in vivo* effects of pVHL (1–157) on TCF/LEF. To this end, we employed zebrafish embryos as *in vivo* models to assess the impact of pVHL (1–157) on Tcf/Lef activity. Ectopic expression of VP16-Tcf7l1ΔN in zebrafish embryos led to a dorsalized phenotype via induction of maternal β-catenin/Tcf activity [19, 25]. The embryos exhibited a markedly expanded expression domain of dorsal marker genes, such as *goosecoid* (*gsc*) and *chordin* (*chd*), two maternal β-catenin/Tcf target genes, at 4.3 hpf (Fig. 6A). With the process of development, the embryos showed “elongated” shape at 12.5 hpf (Fig. 6B). Co-injection of *VHL* mRNA with *vp16-tcf7l1ΔN* mRNA blocked VP16-Tcf7l1ΔN-induced dorsalizing activity in zebrafish embryos (Fig. 6A and B). Indeed, pVHL (1–157) and WT pVHL had a comparatively inhibitory effect on the VP16-Tcf7l1ΔN-induced maternal β-catenin/Tcf action (Fig. 6A and B). These results indicated that the exogenous expression of both pVHL and pVHL (1–157) antagonizes the action of VP16-Tcf7l1ΔN in zebrafish embryos. We next investigated whether pVHL or pVHL (1–157) played a similar physiological role in inhibiting maternal β-catenin/Tcf activity in zebrafish embryos. Forced expression of pVHL markedly decreased the expression range of *gsc* and *chd* at 4.3 hpf (Fig. 6C). Subsequently, the embryos exhibited a phenotype of reduced head tissue and enlarged ventral tissue at 24 hpf (Fig. 6D). Likewise, overexpression of pVHL (1–157) also inhibited maternal β-catenin/Tcf action in zebrafish embryos, leading to a ventralized phenotype (Fig. 6C and D). Taken together, these results indicate that pVHL (1–157) is sufficient to inhibit Tcf/Lef action, thereby resulting in the ventralization of embryos *in vivo*.

### pVhl functions upstream of Tcf7l2 to support dHb neuron differentiation

We next aimed to utilize a genetically inactivated animal model to investigate the physiological role of pVHL in TCF/LEF protein degradation. However, *Vhl*-knockout mice were embryonically lethal and died between E11.5 and 12.5 because of hemorrhagic lesions in the placenta [49]. This limitation hindered our ability to investigate the physiological role of pVHL in TCF/LEF protein degradation. To further elucidate the *in vivo* effects of pVHL on TCF/LEF protein degradation, we used a zebrafish *vhl*-null line, which was generated by a CRISPR/Cas9-based gene editing approach [20]. Analysis of previous RNA-seq data showed that the transcripts of *vhl* were detected from 0 to 96 hpf (Fig. S9A) [50]. Additionally, whole-mount *in situ* hybridization analysis indicated that the *vhl* signals were enriched in the eyes and brain from 24 to 96 hpf (Fig. S9B). Wnt/β-catenin signaling influences axis formation, including anteroposterior, dorsoventral, and left–right body axis, in vertebrates [51]. The *vhl*-null mutant embryos were morphologically indistinguishable from their sibling embryos at 48 hpf, while a slightly reduced anterior head end in *vhl*^−/−^ mutant embryos was observed at 96 hpf (Fig. S10A and B). Hence, genetic deletion of *vhl* does not affect the formation of dorsoventral or anteroposterior axis.

Activation of Wnt/β-catenin signaling in the gastrulation or mid-somite stage disrupts the laterality of Nodal pathway expression in both the LPM and brain [52, 53]. Therefore, left–right asymmetry and laterality of the heart, visceral organ, and brain were examined. Almost all of the offspring of *vhl* heterozygous mutant showed normal expression of heart marker *cmlc2* at 28 and 52 hpf (Fig. S11A and B), normal expression of liver and pancreas marker *foxa3* at 48 hpf (Fig. S11C), and normal expression of liver marker *cp* at 48 hpf (Fig. S11D). Consistently, the expressions of Nodal ligand *spaw* in the lateral plate mesoderm (LPM), Nodal target genes *lefty1* and *pitx2* in the epithalamus, *pitx2* in the posterior left LPM, and *lefty2* in the heart primordia were not altered at the 23-somite stage (Fig. S11E-G). Therefore, genetic deletion of *vhl* does not affect the expression of left-side Nodal signaling and subsequent organ positioning.

**Fig. 7:**
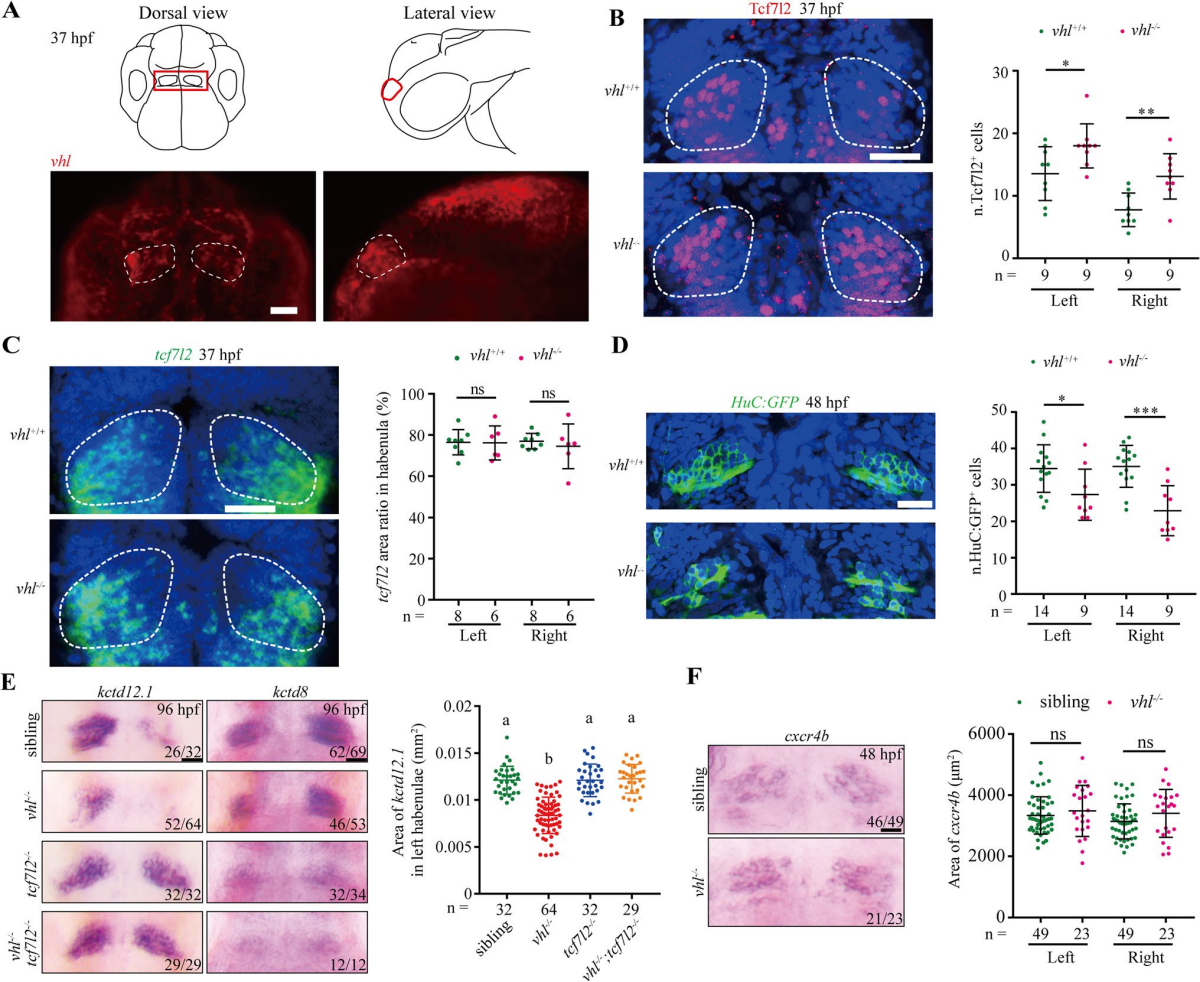
Genetic deletion of *vhl* reduces development of habenular neurons and acts upstream of *tcf7l2*-null mutation (**A**) Diagram of the habenula nucleus localization pattern in the zebrafish head and fluorescence *in situ* hybridization of *vhl* mRNA in the anterior region of zebrafish embryos at 37 hpf. Dorsal view with anterior side upward (left panel) and lateral view with anterior side leftward (right panel). The habenular region is encircled. Scale bar = 50 μm. (**B**) Immunostaining of Tcf7l2-expressing cells in dHb neurons in WT and *vhl*-null embryos at 37 hpf. Dorsal view with anterior side upward. Nuclei are counterstained with DAPI (blue), and the habenular region is encircled. Maximum intensity projection of Z-stack images, which were acquired every 2 μm. Scale bar = 25 μm. Graph shows the number of Tcf7l2-expressing cells in dHb neurons (right panel). The total embryo numbers are given below the X-axis. Values are mean ± S.D. Unpaired *t*-test. *p < 0.05; **p < 0.01. (**C**) Fluorescent RNA probe-labeled *tcf7l2*-expressing cells in dHb neurons in WT and *vhl*-null embryos at 37 hpf. Dorsal view with anterior side upward. Nuclei are counterstained with DAPI (blue), and the habenular region is encircled. Maximum intensity projection of Z-stack images, which were acquired every 2 μm. Scale bar = 25 μm. Quantification of the *tcf7l2*-expressing area in dHb neurons (right panel). The total embryo numbers are given below the X-axis. Values are mean ± S.D. Unpaired *t*-test. ns, not significant. (**D**) Habenular neurons of WT and *vhl*-null embryos in *Tg (huc:GFP)* transgenic background at 48 hpf. Dorsal view with anterior side upward. Nuclei are counterstained with DAPI (blue). Scale bar = 25 μm. Graph shows the number of left and right lateral HuC:GFP^+^ habenular neurons (right panel). The total embryo numbers are given below the X-axis. Values are mean ± S.D. Unpaired *t*-test. *p < 0.05; ***p < 0.001. (**E**) Expression of *kctd12.1* and *kctd8* in dHb in embryos with indicated genotypes at 96 hpf. *kctd12.1*, which is reduced in *vhl*-null embryos, is enhanced in *tcf7l2*-null mutants, and *kctd8*, which is less strongly reduced in *vhl*-null embryos, is absent in *tcf7l2*-null mutants; *vhl/tcf7l2* double mutants show the same character as that of *tcf7l2*-null mutants. Dorsal view with anterior side upward. Scale bar = 20 μm. Quantification of the *kctd12.1*-expressing area in left lateral habenular (right panel). The total embryo numbers are given along the X-axis. Values are mean ± S.D. One-way ANOVA analysis with Tukey’s post-hoc test. Different letters indicate significant differences (*p* < 0.0001). (**F**) The expression of *cxcr4b* in dHb neurons in sibling and mutant embryos at 48 hpf. Scale bar = 20 μm. Quantification of the *cxcr4b*-expressing area in dHb neurons (right panel). The total embryo numbers are given below the X-axis. Values are mean ± S.D. Unpaired *t*-test. ns, not significant

We noted *vhl* was enriched on the left and right sides of dHb neurons at 37 hpf (Fig. 7A and Fig. S9B). Additionally, the zebrafish *vhl*-null mutant embryos exhibit increased Tcf7l2 protein levels. In particular, Wnt ligands, Axin1/2, and Tcf7l2 are expressed in the diencephalon and regulate dHb development [52, 54, 55]. Therefore, we next tested whether depletion of pVhl affects Tcf7l2 protein levels in dHb and habenular neuron development in zebrafish embryos. Indeed, Tcf7l2 also expressed on the left and right sides of dHb neurons at 37 hpf, which is immediately after initial Tcf7l2 protein expression in dHb neurons [55]. Hence, pVhl may regulate the expression of Tcf7l2 at this stage during embryogenesis. We therefore monitored the expression of Tcf7l2 on the left and right sides of dHb neurons at 37 hpf. The *vhl*-null mutant embryos exhibit significantly increased numbers of Tcf7l2^+^ cells while unchanged *tcf7l2* mRNA levels on both the left and right sides of dHb neurons (Fig. 7B and C), further suggesting that depletion of pVhl increases stability of Tcf7l2 protein. Moreover, we observed that, at 48 hpf, depletion of pVhl markedly reduced the numbers of GFP^+^ cells in *vhl-*null mutant embryos with a *Tg (huc:GFP)* transgenic background (Fig. 7D). This phenotype did not result from the growth retardation since body length, the quantitative indicator of developmental rate, had no differences between the WT and *vhl*-null groups (Fig. S10A and B). We noticed that this phenotype is reminiscent of embryos after premature activation of Wnt/β-catenin signaling between 24 to 26 hpf, which also delays habenular neuron differentiation with reduced numbers of GFP^+^ cells in embryos at this stage [56].

The dHb, which comprises left–right asymmetric lateral (dHbl) and medial (dHbm) subnuclei, requires Wnt signaling for the proper determination of dHbl neuron fate [56]. The increased expression of Tcf7l2 protein in dHb neurons of *vhl*-null zebrafish may function in premature activation of Wnt/β-catenin signaling and then lead to delayed habenular neuron differentiation. To test this, we examined the epistatic relationship between null mutations in *vhl* and *tcf7l2* by inspecting the expression of dHbl marker *kctd12.1* and dHbm marker *kctd8* in progeny embryos of *vhl/tcf7l2* double heterozygous mutants at 96 hpf. Compared with that in WT sibling embryos, expression of *kctd12.1* was reduced, while that of *kctd8* was less strongly reduced in *vhl*-null embryos (Fig. 7E), which was consistent with expression in 96 hpf embryos treated with LiCl or BIO between 24 and 26 hpf [56]. Consistent with the findings of a previous study [55], *tcf7l2*-null mutant embryos showed enhanced expression of *kctd12.1* and reduced expression of *kctd8* (Fig. 7E). Considering that pVhl promotes Tcf7l2 proteasomal degradation, pVhl should act upstream of Tcfl2. If the phenotype of *vhl*^*−/−*^ mutants depends on enhanced expression of Tcf7l2 protein, the reduced *kctd12.1* expression and less strongly reduced *kctd8* expression should not be observed in *vhl/tcf7l2* double mutants, instead of the phenotype of *tcf7l2* single mutant. In other words, the expression of *kctd12.1* and *kctd8* in a *tcf7l2*^*−/−*^ background should be enhanced and reduced respectively, irrespective of the presence or absence of pVhl function. As expected, the expression of *kctd12.1* and *kctd8* in *vhl/tcf7l2* double mutants was consistent with that in *tcf7l2* single mutant embryos (Fig. 7E). We quantified the expression area of left lateral *kctd12.1* and significant reduction was only observed in *vhl* single mutant embryos (Fig. 7E). Additionally, it has been reported that Wnt/β-catenin signaling is involved in the generation of habenular precursor cells [55]. We, therefore, investigated the expression of *cxcr4b*, a marker of habenular progenitor cells, in the *vhl* mutant embryos at 48 hpf. Indeed, we did not observe any alternation of *cxcr4b* expression in the diencephalon of *vhl* mutant embryos (Fig. 7F). This result suggested that the phenotypes in *vhl*^*−/−*^ mutants are not from the reduction of precursor cells.

Collectively, these results suggested that pVhl destabilizes Tcf7l2 in habenula as well as acts upstream of Tcf7l2 and plays a specific role in dHb development.

## Discussion

The TCF/LEF transcription factor family includes four members that display distinct and sometimes redundant functions. In this study, we discovered that pVHL directly interacts with all four members of the TCF/LEF family and promotes their degradation via a ubiquitin-independent proteasomal pathway. Through this mechanism, pVHL modulates the abundance of TCF/LEF proteins, thereby playing a critical role in fine-tuning the nuclear output of the Wnt/β-catenin signaling cascade with precise spatial and temporal regulation. The naturally occurring, cancer-associated truncated mutant pVHL (1–157), which functions independently of the VBC complex, suppressed the proliferation of cultured HCT116 colon cancer cells and disrupted dorsoventral axis specification during zebrafish embryogenesis. Using *vhl-* and *tcf7l2-*knockout zebrafish embryos, we found that pVhl functions in modulation of the protein stability of Tcf7l2 in habenular neurons as well as in regulation of development of dorsal habenular neurons.

The *VHL* gene is inactivated in at least two-thirds of sporadic ccRCC cases [57, 58]. Wnt/β-catenin signaling is constitutively active in RCC; however, the molecular mechanisms underlying its regulation remain largely elusive [59]. pVHL targets TCF/LEF for degradation. Specifically, our results demonstrated that reintroducing pVHL in a pVHL-deficient ccRCC cell line, 786-O, reduces its responsiveness to Wnt signaling activation. This indicates that the loss of pVHL likely induces a Wnt-hypersensitive state through the accumulation of TCF/LEF proteins, thereby enhancing Wnt signaling and facilitating the execution of oncogenic gene expression programs, which in turn contribute to elevated cancer susceptibility.

An earlier study suggested that the promoter of *VHL* responds to β-catenin/TCF7L2 and that pVHL has interplay with the Wnt/β-catenin pathway during colorectal tumorigenesis [60]. Our findings reveal a mechanistic link between these two critical signaling pathways and may contribute to a deeper understanding of the interplay between the pVHL and Wnt/β-catenin signaling pathways in cancer pathogenesis. In addition, a previous study showed that in colon cancer, pVHL may be an oncogenic protein because it promotes proteasomal degradation of transcription factor Krüppel-like factor 4 (KLF4), which is involved in regulation of cell-fate decision as a tumor suppressor [61]. Here, we found that pVHL promotes proteasomal degradation of transcription factor TCF/LEF and inhibits oncogenic Wnt/β-catenin signaling in HCT116 colorectal carcinoma cells. However, reduction of TCF7L2 decreased transcriptional levels of *Klf4* in Wnt overactivated colon tumors of mice [62]. In addition, distinct TCF/LEF members were indeed regulated differentially in colon tumorigenesis [62–65]. Thus, the regulation of KLF and TCF/LEF by pVHL, as well as the role of pVHL in colon tumors, appears to be complex. Further investigation is warranted to elucidate the physiological impact of pVHL across different types of colon cancer.

The Wnt/β-catenin signaling pathway plays a crucial role in the formation of the body axis in vertebrates. In zebrafish embryos, the Wnt/β-catenin signaling pathway regulates the establishment of the dorsoventral, anteroposterior, and left–right body axes [51]. In our study, the overexpression of pVHL and pVHL (1–157) suppressed the expression of the dorsal marker genes *gsc* and *chd*, leading to ventralized phenotypes in zebrafish embryos. However, we did not observe any deficiency of axis formation in *vhl*-null mutant embryos. A possible explanation for this observation is that the expression levels of *vhl* are not sufficient to influence the formation of axis. Another, maternally deposited mRNA or protein might contribute this phenotype. Zebrafish genome harbors two *vhl* genes, *vhl* and *vhlb* (Fig. S7A). Both are functional pVhl [66]. Previous studies reported that premature termination codons in zebrafish mutants can activate a compensation mechanism by upregulating the expression of other family members [67, 68]. Hence, pVhlb might function redundantly and/or compromise the genetic deletion of *vhl*. Interestingly, we observed that *vhl* mRNA was highly enriched in the zebrafish habenula at 37 hpf, shortly after the initial appearance of Tcf7l2 protein in dorsal habenular neurons. pVhl regulates the protein stability of Tcf7l2 and promotes the development of dHb neurons, as evidenced by a reduction in HuC:GFP^+^ cell numbers and diminished expression of dHbl and dHbm markers following *vhl* knockout, without influencing left–right patterning. In contrast, *axin1* mutants exhibited bilateral Nodal gene expression in the epithalamus but maintained normal organ positioning [52]. Notably, the above phenotype in *vhl*-null mutants produces phenocopied embryos after treatment with LiCl or BIO between 24 and 26 hpf, leading to premature activation of Wnt signaling [56]. Alternatively, this phenotype may arise from the initial increased Tcf7l2 expression in dHb neurons, which causes temporally and locally increased Wnt activity. Indeed, the *vhl*-null mutation is upstream of functional Tcf7l2, as shown by an epistatic analysis between *vhl*-null and *tcf7l2*-null mutations. During habenula development, Wnt/β-catenin signaling functions from cellular proliferation to the establishment and maintenance of cell identity. In addition, Wnt/β-catenin signaling also functions at multiple stages in these processes [52, 54–56, 69]. This makes the complexity of the output effects of this signaling pathway in regulation of habenula development [69]. Future studies will be needed to determine the comprehensive developmental basis underlying the phenotype after depletion of zebrafish pVhl.

As mentioned earlier, TCF/LEF family in vertebrates contains four members. Disruption of any one results in a distinct phenotype [9]. Indeed, knockout of *Tcf/Lef* genes in mouse studies demonstrates both redundant and non-redundant functions during embryonic development and adult stem cell regulation [70]. Different member mediates tissue- and stage-specific Wnt regulation in these processes. In particular, TCF7L1 appears to function through both β-catenin-dependent and β-catenin-independent interactions in different developmental contexts [71]. Given that pVHL has the potential to promote the degradation of all four TCF/LEF members, future efforts are needed to investigate the potential physiological effect of interaction between pVHL and distinct members of TCF/LEF family in different cellular contexts. Furthermore, in addition to the TCF/LEF factors that we have identified, there may be other substrates mediated by pVHL and directly degraded by the 26S proteasome. The efforts to determine that pVHL mediates substrate degradation by direct interaction and complexation with the 26S proteasome should have broad utility for better understanding the physiological role and molecular function of pVHL in the future.

## Supplementary Information

Below is the link to the electronic supplementary material.

Supplementary file1 Generation of *VHL*-null cell lines (**A**) Schematic illustrations of genomic structures and target positions of CRISPR/Cas9-mediated *VHL* mutation. TSS denotes translation start codon; the black box denotes exon; gray box denotes UTR; black lines denote introns. The length of each exon is shown.* VHL* knockout clones #1-4 were generated via single-target editing (Tg1), whereas clone #5 was established through dual-target knockout (Tg1 & Tg2). (**B**) Schematic illustrations of pVHL truncated protein structures. Two Met denote different translation start codons in pVHL. Numbers denote amino acid positions of critical domain and mutant protein length (TIF 13683 KB)

Supplementary file2 The overexpression of pVHL reduced the abundance of Tcf/Lef (**A**,**B**) Exogenous Tcf/Lef protein levels in control or pVHL-overexpressing HEK293T and HCT116 cells (TIF 12359 KB)

Supplementary file3 TCF/LEF amino acid sequence alignment Amino acid sequence alignment of human, mouse, *Xenopus*, and zebrafish TCF/LEF. Conserved lysines are indicated in red. The β-catenin-binding domain is indicated in brilliant blue. The HMG is indicated in deep blue. The NLS is indicated in orange. Accession numbers are: human TCF7 NP_003193.2, mouse TCF7 NP_001300910.1, zebrafish Tcf7 NP_001012389.1, *Xenopus *Tcf7 NP_989421.1, human TCF7L1 NP_112573.1, mouse TCF7L1 NP_001073290.1, zebrafish Tcf7l1a NP_571344.1, zebrafish Tcf7l1b NP_571371.2, *Xenopus *Tcf7l1 NP_001005640.1, human TCF7L2 NP_001139746.1, mouse TCF7L2 NP_001136390.1, zebrafish Tcf7l2 NP_571334.1, *Xenopus *Tcf7l2 NP_001231922.1, human LEF1 NP_057353.1, mouse LEF1 NP_034833.2, zebrafish Lef1 NP_571501.1 and *Xenopus Lef1 *NP_001230763.1 (TIF 323754 KB)

Supplementary file4 Mapping the binding domains of TCF7L2 to pVHL (**A**, **B**) Mapping TCF7L2 binding domain associated with pVHL in transfected HEK293T cells by Co-IP assay. Red asterisk indicates the specific band. (**C**) Subcellular localization of HA-tagged TCF7L2 mutants in HeLa cells. Scale bar =10 μm. (**D**) Tcf7l1-HMG DBD protein levels in HEK293T cells overexpressing Flag-pVHL. (**E**) Genetic deletion of *VHL* has minimal impact on TCF7L2 binding to the *AXIN2* and *NKD1* promoters in HEK293T cells, as determined by ChIP-qPCR. Similar results were obtained from three experiments. Values are mean ± S.D. Unpaired *t*-test. ns, not significant (TIF 62937 KB)

Supplementary file5 The effects of various treatment on the protein levels of TCF/LEF (**A**) *Xenopus* Tcf7l2 protein levels in HEK293T cells with pVHL- or pVHL-S111C/H115N/W117R-overexpression. (**B**) pVHL promoted endogenous TCF7L2 degradation in presence of the DMOG. Western blot analysis of WCL derived from HEK293T cells transfected with indicated plasmid DNA and either untreated or treated with 200 μM DMOG for 12 h. (**C**) Time-course for non-phospho-(active) β-catenin, β-catenin, and TCF7L2 protein levels in starved HEK293T cells. Western blot analysis of WCL derived from starved HEK293T cells at indicated time points (0, 2, 4, 8 h) (TIF 4073 KB)

Supplementary file6 HIF activity did not upregulate the protein levels of TCF7, TCF7L1, and TCF7L2. (**A**) Endogenous TCF7, TCF7L1, TCF7L2, and HIF-1α protein levels under normoxic (21% O_2_) or hypoxic (1% O_2_) conditions for 24 h in HEK293T cells. (**B**) Schematic illustrations of genomic structures and target positions of CRISPR/Cas9-mediated *HIF1-β* mutation. TSS denotes translation start codon; the black box denotes exon; gray box denotes UTR; black lines denote introns. (**C**) Schematic illustrations of HIF1-β truncated protein structures. Numbers denote amino acid positions of critical domain and mutant protein length. (**D**) Endogenous TCF7, TCF7L1, TCF7L2, and HIF-1α protein level under normoxic (21% O_2_) or hypoxic (1% O_2_) condition for 24 h in *HIF-1β*-knockout HEK293T cells (TIF 36803 KB)

Supplementary file7 The gene structure and amino acid sequence of pVHL and pVHLL (**A**) Schematic representation of human and zebrafish *VHL/vhl(b)* or *VHLL* gene structure. TSS denotes transcriptional start site. Exons are shown as black boxes, and UTR are shown as gray boxes. Introns are shown as lines. The length of each exon is shown. (**B**) Amino acid sequence alignment of human pVHL and pVHLL, as well as zebrafish pVhl and pVhlb. The key residues for prolyl hydroxylation binding were highlighted with red color. The Elongin C binding domain was highlighted with a blue box. Accession numbers are: human pVHL NP_000542.1, human pVHLL NP_001004319.1, zebrafish pVhl NP_001074153.1, zebrafish pVhlb NP_001122264.1. (**C**) Co-IP assay revealed the interaction between endogenous TCF7L2 and transfected Flag-pVHLL in HEK293T cells. (**D**) Endogenous TCF protein levels in HEK293T cells with increasing Flag-pVHLL overexpression (TIF 47357 KB)

Supplementary file8 The pVHL (1-157) is sufficient to suppress cellular proliferation in HCT116 cells Cell proliferation in HCT116 cells was assessed by EdU assays. Following transfection with mCherry (control), Flag-pVHL (1-157), or Flag-pVHLL for 24 h, HCT116 cells were incubated with 10 μM EdU for 2 h. Proliferation signals (green) were subsequently detected via EdU staining and co-localized with mCherry or Flag immunofluorescence (red). Nuclei are counterstained with DAPI (blue). Results are derived from three independent experiments with triple replicates. All individual data points are shown. Values are means ± S.D. ns, not significant; **p < 0.01; ****p < 0.0001. Unpaired *t *test, two-tailed. Scale bar=100 μm (TIF 17727 KB)

Supplementary file9 The spatiotemporal expression pattern of zebrafish *vhl *(**A**) The transcriptional levels of *vhl* at the indicated embryonic stages from a published RNA-seq data. Numbers indicate different developmental stages as hpf. (**B**) Whole-mount *in situ* hybridization analysis of zebrafish *vhl* mRNA at the indicated stages. All panels are dorsal, top, or lateral views with animal pole up or anterior to the left. The red arrowhead and white dashline indicated the habenulae in lateral and dorsal view respectively. Scale bars = 200 μm (TIF 28522 KB)

Supplementary file10 Representative morphologies of *vhl*, *tcf7l2*, and *vhl/tcf7l2* double mutants (**A**, **B**) Representative images of mutant embryos at 48 hpf (**A**) and 96 hpf (**B**) with indicated genotypes. Scale bar= 250 μm. Quantification of the body length of a-b (mouth to end of tail through center of ear vesicle) in sibling and *vhl* mutant embryos at 48 hpf (**A**, right panel). And quantification of the head length of a-b (mouth to center of ear vesicle) and trunk length of b-c (center of ear vesicle to end of tail) in sibling and *vhl* mutant embryos at 96 hpf (**B**, right panel). The total embryo numbers are given below the X-axis. Values are mean ± S.D. Unpaired *t*-test. ns, not significant; ** *p *< 0.01 (TIF 6504 KB)

Supplementary file11 Depletion of pVhl had little effect on left-right asymmetric development (**A**-**G**) Offspring embryos of heterozygous *vhl *mutants are examined for *cmlc2* expression at 28 hpf (**A**) and 52 hpf (**B**), *foxa3* expression at 48 hpf (**C**), *cp* expression at 48 hpf (**D**), *spaw* expression at the 23-somite stage (**E**), *lefty1/lefty2* expression at the 23-somite stage (**F**), *pitx2* expression in head (left) and LPM (right) at the 23-somite stage (**G**). Embryos are shown in ventral (**A**, **B**) or dorsal view (**C**-**G**) with anterior side upward. The red asterisk indicates the expression of *lefty1* in the diencephalon, and the black arrow indicates the expression of *lefty2* in heart field (**F**). l, liver; p, pancreas; g, gut. Embryos are from at least three pairs of adult fish. Scale bar = 100 μm (TIF 13002 KB)

Supplementary file12 (PDF 117 KB)

Supplementary file13 (PDF 144 KB)

## Data Availability

All the data are within the article and supporting information. All the data are to be shared upon request (Xiaozhi Rong, Ocean University of China, rongxiaozhi@ouc.edu.cn).
