# Supplementary figures and images for: pVHL regulates protein stability of the TCF/LEF transcription factor family via ubiquitin-independent proteasomal degradation
Source: Cell Mol Life Sci. 2025 Sep 4;82(1):335. doi: 10.1007/s00018-025-05852-0 (PMC12411348; doi:10.1007/s00018-025-05852-0)

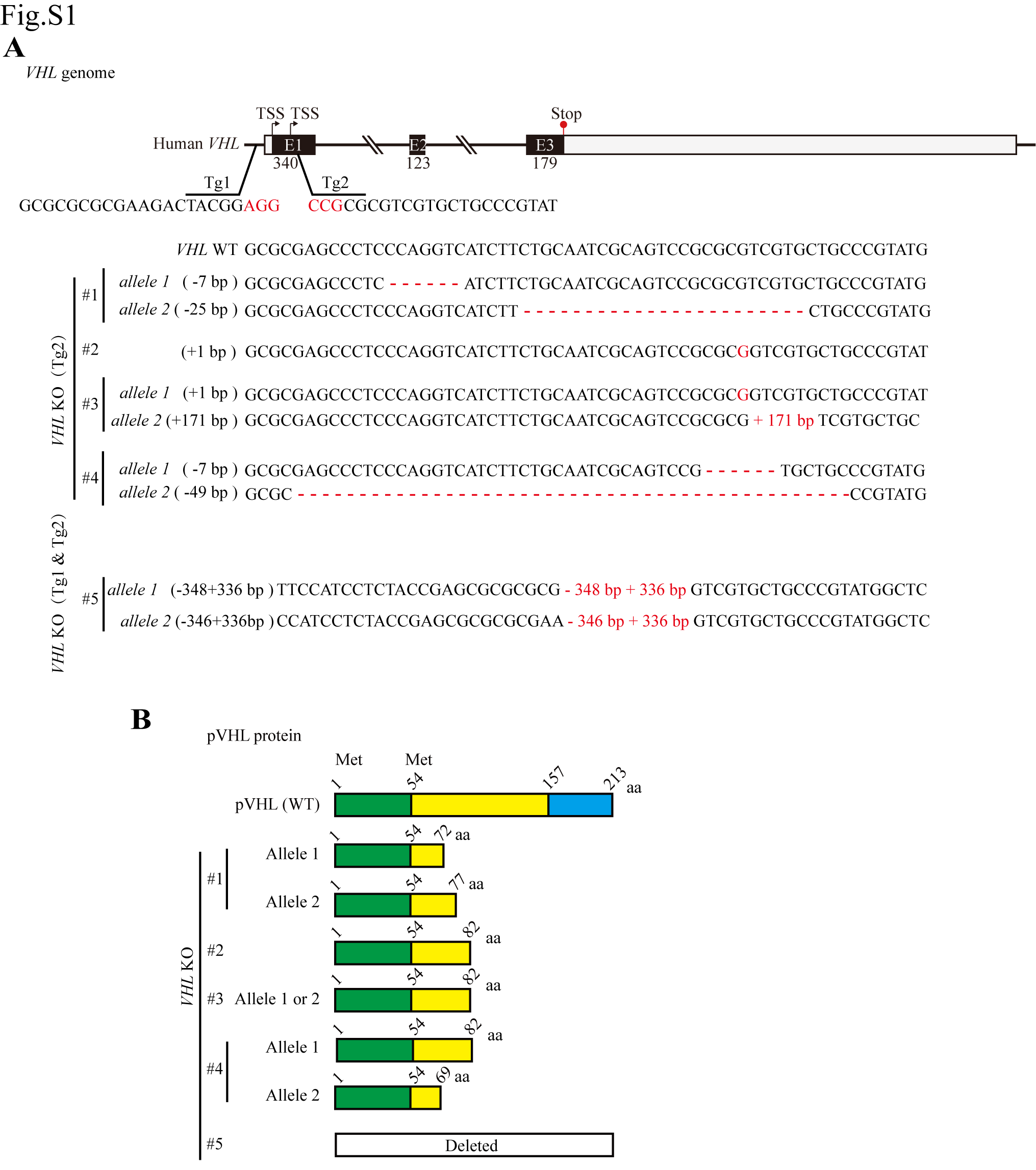

Supplement: Supplementary file 1 — Supplementary file1 Generation of VHL-null cell lines (A) Schematic illustrations of genomic structures and target positions of CRISPR/Cas9-mediated VHL mutation. TSS denotes translation start codon; the black box denotes exon; gray box denotes UTR; black lines denote introns. The length of each exon is shown. VHL knockout clones #1-4 were generated via single-target editing (Tg1), whereas clone #5 was established through dual-target knockout (Tg1 & Tg2). (B) Schematic illustrations of pVHL truncated protein structures. Two Met denote different translation start codons in pVHL. Numbers denote amino acid positions of critical domain and mutant protein length (TIF 13683 KB) [file 18_2025_5852_MOESM1_ESM.tif]

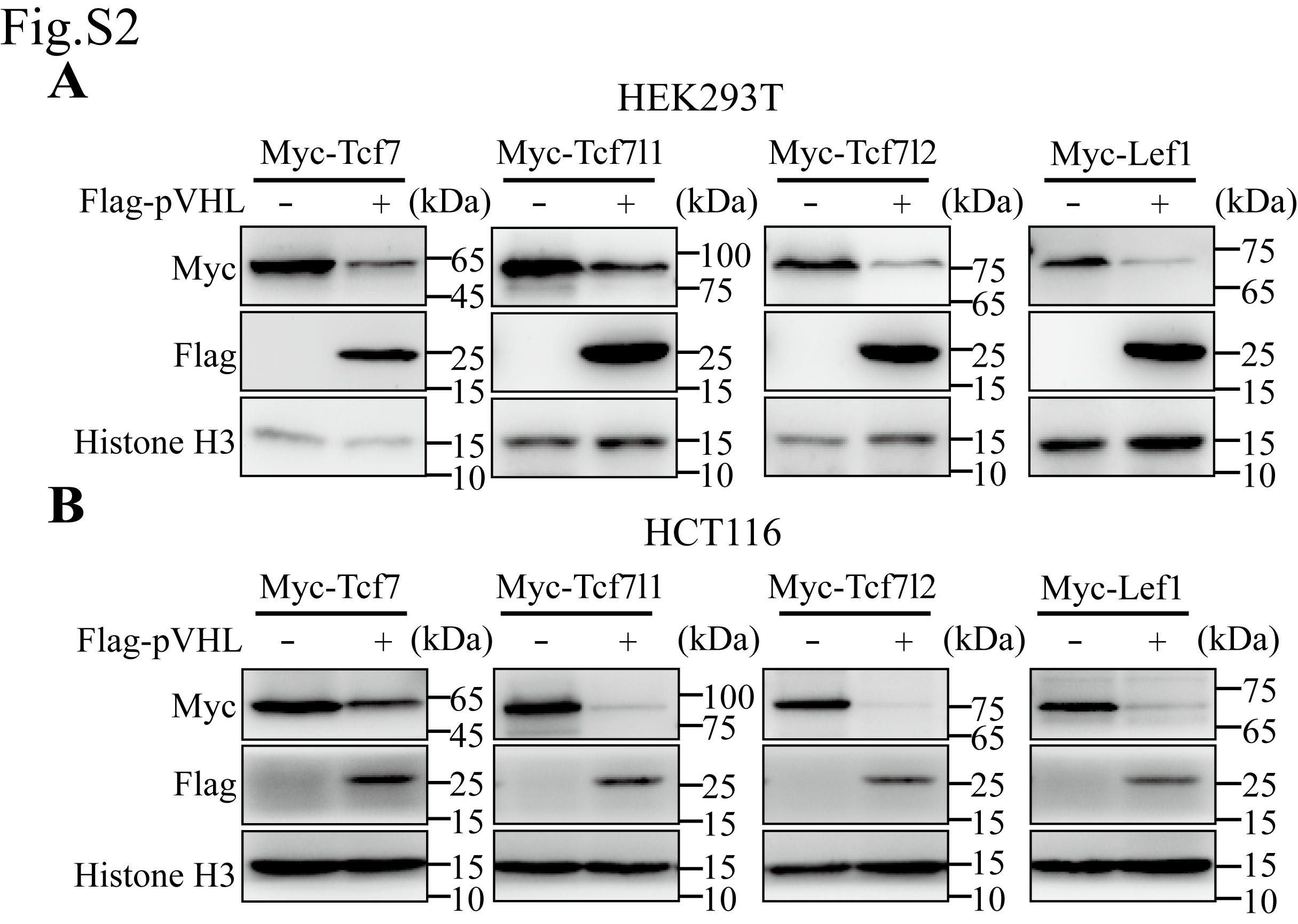

Supplement: Supplementary file 2 — Supplementary file2 The overexpression of pVHL reduced the abundance of Tcf/Lef (A,B) Exogenous Tcf/Lef protein levels in control or pVHL-overexpressing HEK293T and HCT116 cells (TIF 12359 KB) [file 18_2025_5852_MOESM2_ESM.tif]

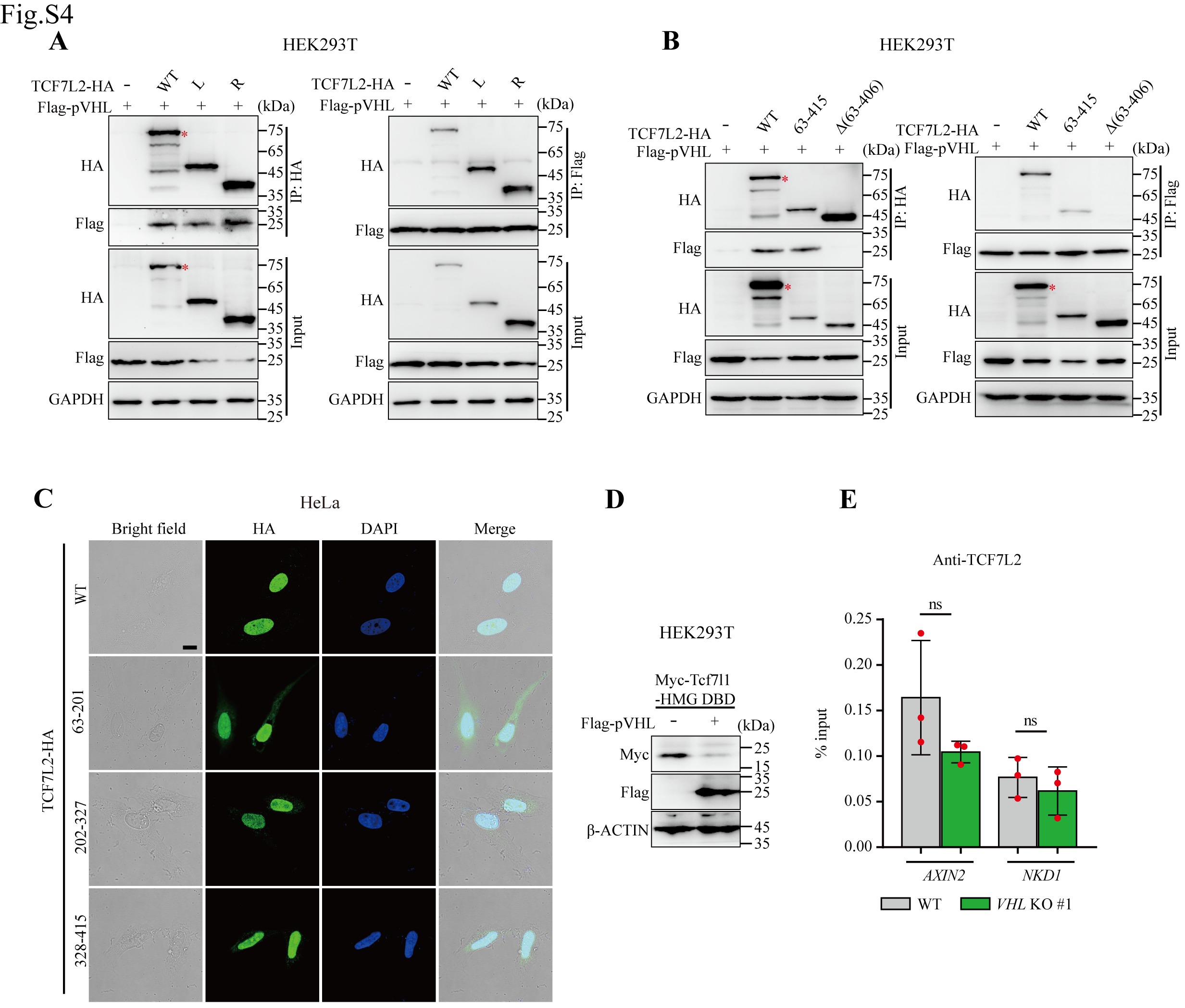

Supplement: Supplementary file 4 — Supplementary file4 Mapping the binding domains of TCF7L2 to pVHL (A, B) Mapping TCF7L2 binding domain associated with pVHL in transfected HEK293T cells by Co-IP assay. Red asterisk indicates the specific band. (C) Subcellular localization of HA-tagged TCF7L2 mutants in HeLa cells. Scale bar =10 μm. (D) Tcf7l1-HMG DBD protein levels in HEK293T cells overexpressing Flag-pVHL. (E) Genetic deletion of VHL has minimal impact on TCF7L2 binding to the AXIN2 and NKD1 promoters in HEK293T cells, as determined by ChIP-qPCR. Similar results were obtained from three experiments. Values are mean ± S.D. Unpaired t-test. ns, not significant (TIF 62937 KB) [file 18_2025_5852_MOESM4_ESM.tif]

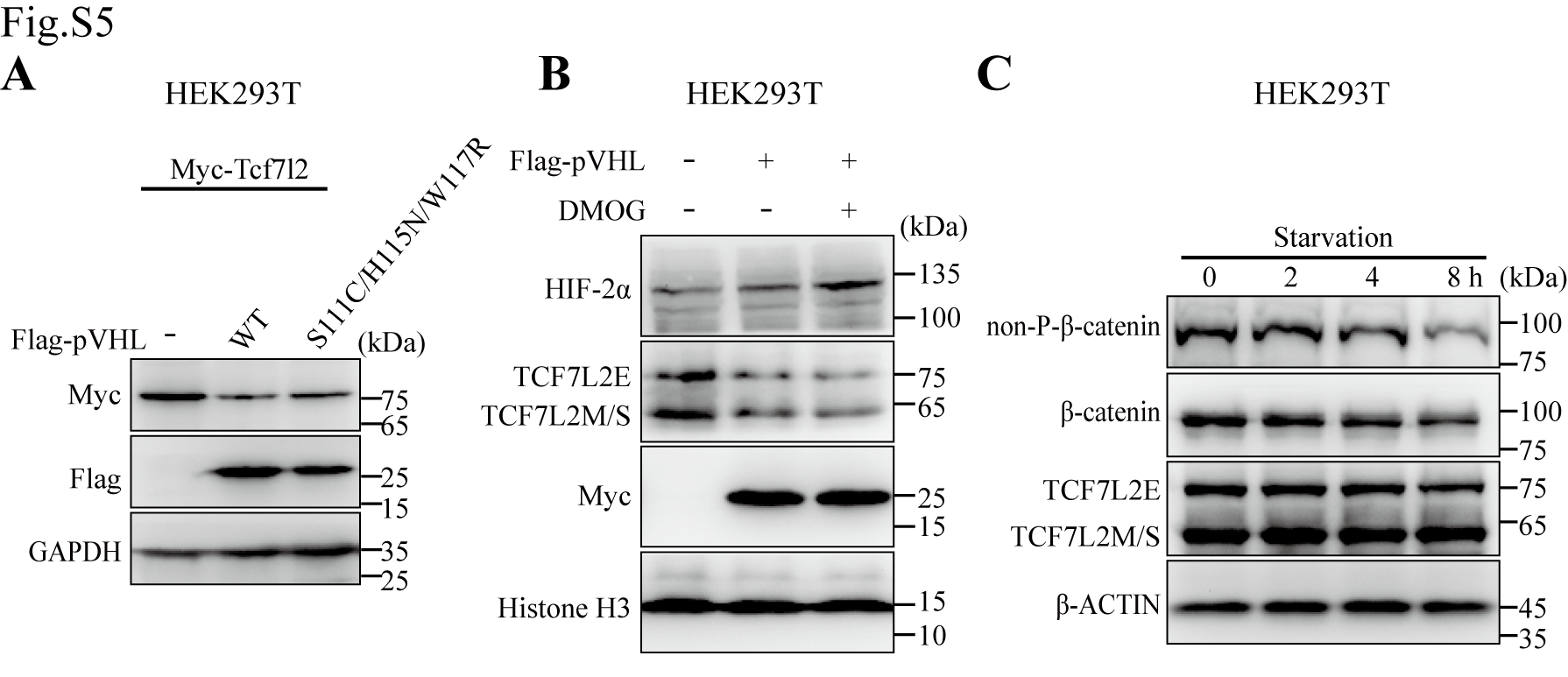

Supplement: Supplementary file 5 — Supplementary file5 The effects of various treatment on the protein levels of TCF/LEF (A) Xenopus Tcf7l2 protein levels in HEK293T cells with pVHL- or pVHL-S111C/H115N/W117R-overexpression. (B) pVHL promoted endogenous TCF7L2 degradation in presence of the DMOG. Western blot analysis of WCL derived from HEK293T cells transfected with indicated plasmid DNA and either untreated or treated with 200 μM DMOG for 12 h. (C) Time-course for non-phospho-(active) β-catenin, β-catenin, and TCF7L2 protein levels in starved HEK293T cells. Western blot analysis of WCL derived from starved HEK293T cells at indicated time points (0, 2, 4, 8 h) (TIF 4073 KB) [file 18_2025_5852_MOESM5_ESM.tif]

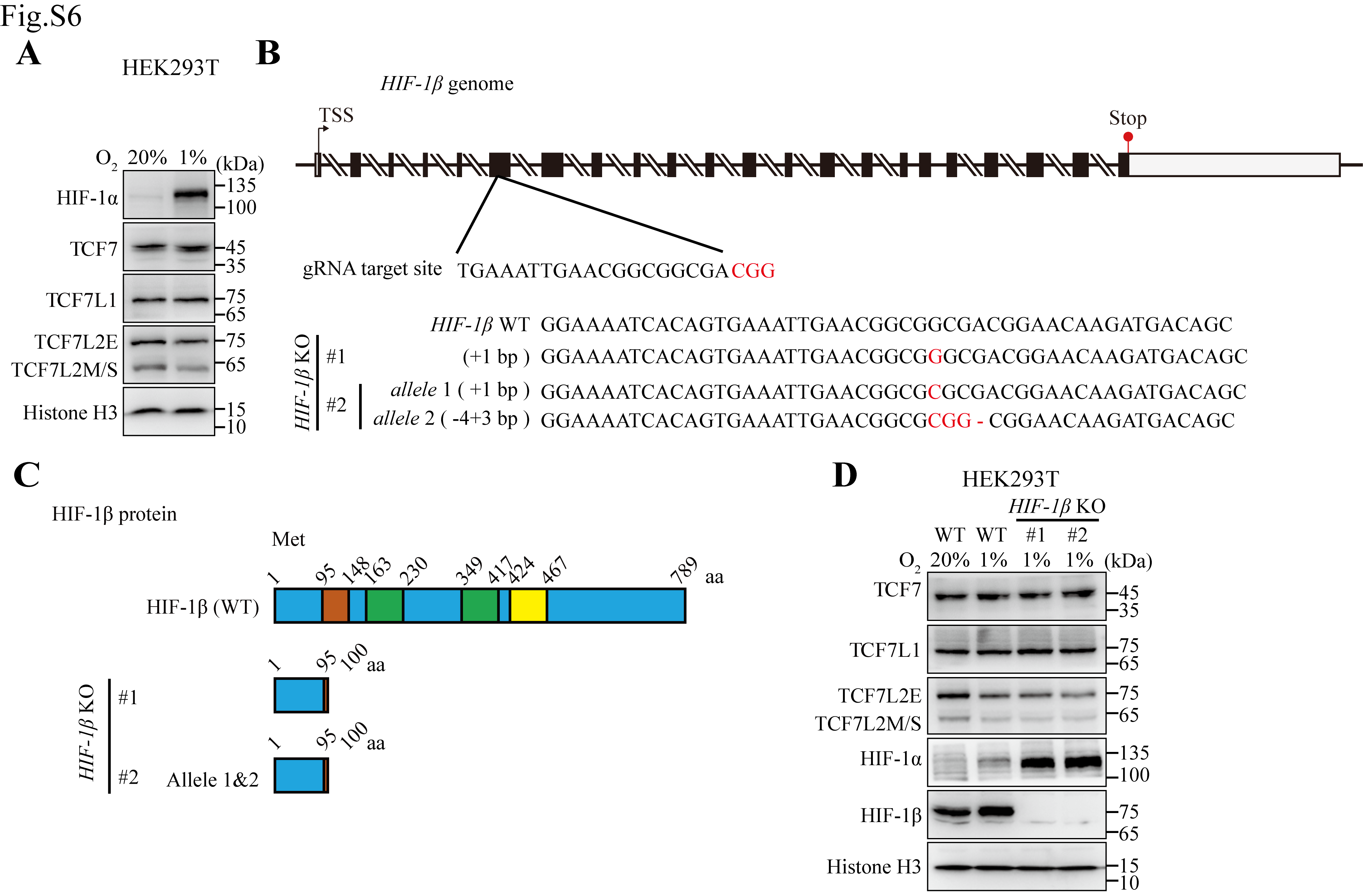

Supplement: Supplementary file 6 — Supplementary file6 HIF activity did not upregulate the protein levels of TCF7, TCF7L1, and TCF7L2. (A) Endogenous TCF7, TCF7L1, TCF7L2, and HIF-1α protein levels under normoxic (21% O2) or hypoxic (1% O2) conditions for 24 h in HEK293T cells. (B) Schematic illustrations of genomic structures and target positions of CRISPR/Cas9-mediated HIF1-β mutation. TSS denotes translation start codon; the black box denotes exon; gray box denotes UTR; black lines denote introns. (C) Schematic illustrations of HIF1-β truncated protein structures. Numbers denote amino acid positions of critical domain and mutant protein length. (D) Endogenous TCF7, TCF7L1, TCF7L2, and HIF-1α protein level under normoxic (21% O2) or hypoxic (1% O2) condition for 24 h in HIF-1β-knockout HEK293T cells (TIF 36803 KB) [file 18_2025_5852_MOESM6_ESM.tif]

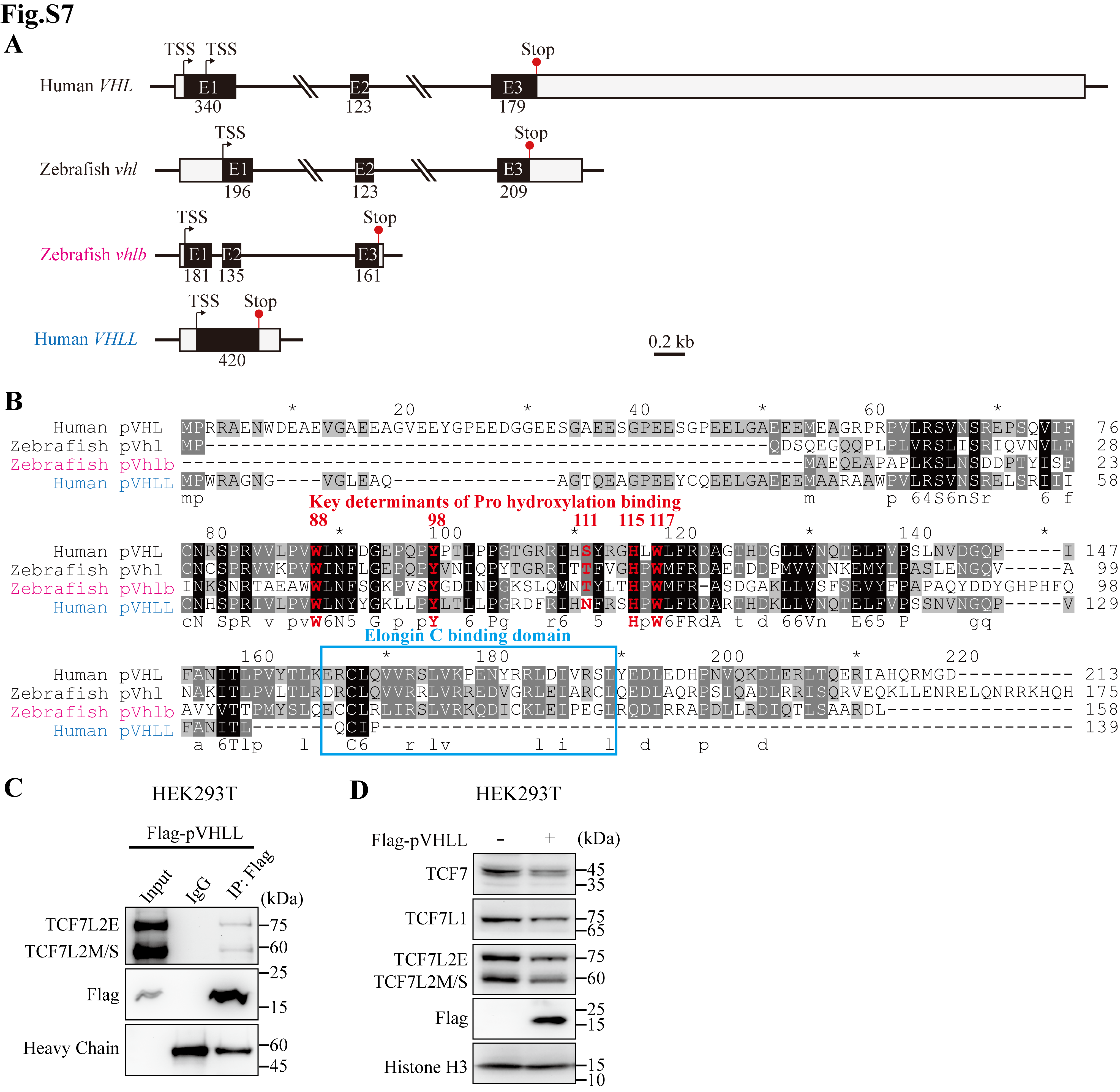

Supplement: Supplementary file 7 — Supplementary file7 The gene structure and amino acid sequence of pVHL and pVHLL (A) Schematic representation of human and zebrafish VHL/vhl(b) or VHLL gene structure. TSS denotes transcriptional start site. Exons are shown as black boxes, and UTR are shown as gray boxes. Introns are shown as lines. The length of each exon is shown. (B) Amino acid sequence alignment of human pVHL and pVHLL, as well as zebrafish pVhl and pVhlb. The key residues for prolyl hydroxylation binding were highlighted with red color. The Elongin C binding domain was highlighted with a blue box. Accession numbers are: human pVHL NP_000542.1, human pVHLL NP_001004319.1, zebrafish pVhl NP_001074153.1, zebrafish pVhlb NP_001122264.1. (C) Co-IP assay revealed the interaction between endogenous TCF7L2 and transfected Flag-pVHLL in HEK293T cells. (D) Endogenous TCF protein levels in HEK293T cells with increasing Flag-pVHLL overexpression (TIF 47357 KB) [file 18_2025_5852_MOESM7_ESM.tif]

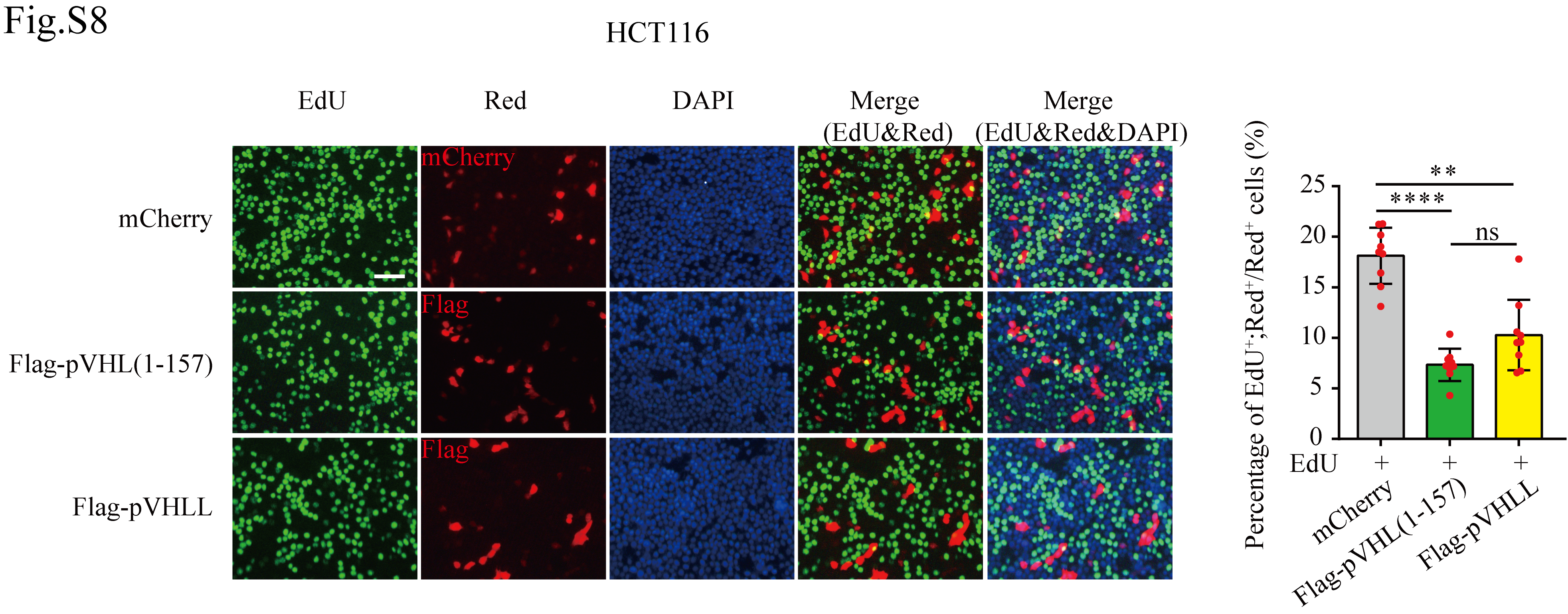

Supplement: Supplementary file 8 — Supplementary file8 The pVHL (1-157) is sufficient to suppress cellular proliferation in HCT116 cells Cell proliferation in HCT116 cells was assessed by EdU assays. Following transfection with mCherry (control), Flag-pVHL (1-157), or Flag-pVHLL for 24 h, HCT116 cells were incubated with 10 μM EdU for 2 h. Proliferation signals (green) were subsequently detected via EdU staining and co-localized with mCherry or Flag immunofluorescence (red). Nuclei are counterstained with DAPI (blue). Results are derived from three independent experiments with triple replicates. All individual data points are shown. Values are means ± S.D. ns, not significant; **p < 0.01; ****p < 0.0001. Unpaired t test, two-tailed. Scale bar=100 μm (TIF 17727 KB) [file 18_2025_5852_MOESM8_ESM.tif]

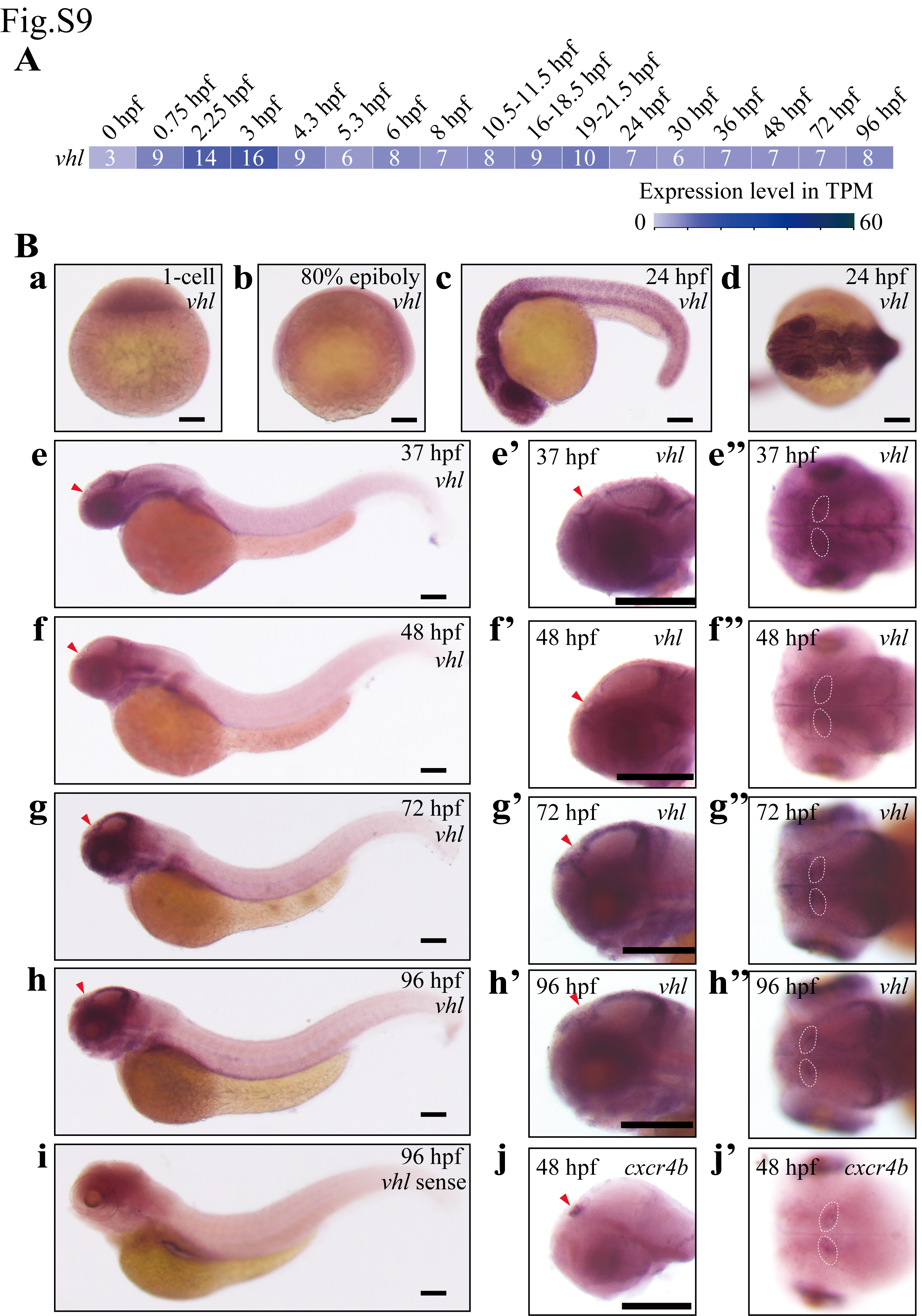

Supplement: Supplementary file 9 — Supplementary file9 The spatiotemporal expression pattern of zebrafish vhl (A) The transcriptional levels of vhl at the indicated embryonic stages from a published RNA-seq data. Numbers indicate different developmental stages as hpf. (B) Whole-mount in situ hybridization analysis of zebrafish vhl mRNA at the indicated stages. All panels are dorsal, top, or lateral views with animal pole up or anterior to the left. The red arrowhead and white dashline indicated the habenulae in lateral and dorsal view respectively. Scale bars = 200 μm (TIF 28522 KB) [file 18_2025_5852_MOESM9_ESM.tif]

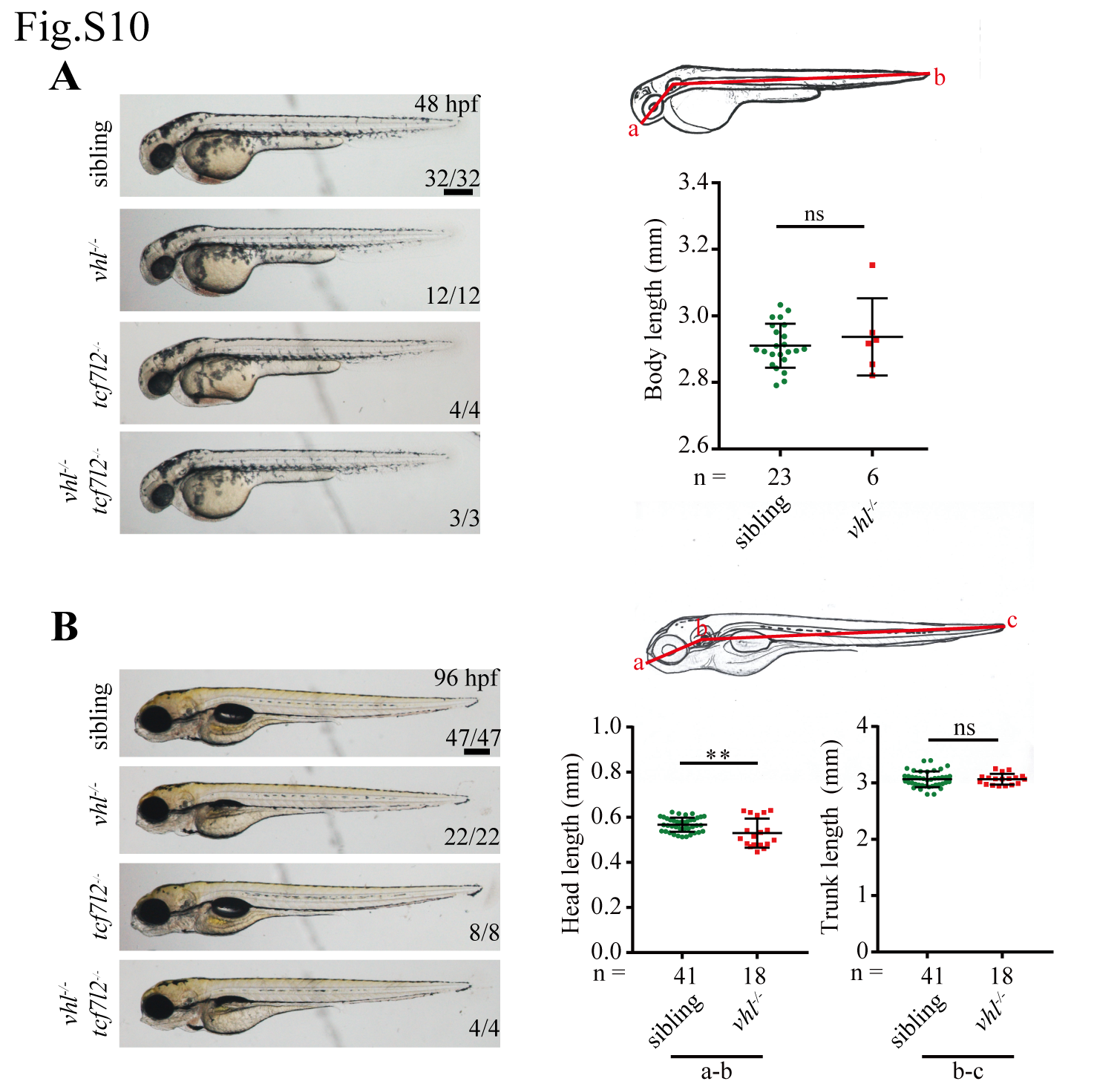

Supplement: Supplementary file 10 — Supplementary file10 Representative morphologies of vhl, tcf7l2, and vhl/tcf7l2 double mutants (A, B) Representative images of mutant embryos at 48 hpf (A) and 96 hpf (B) with indicated genotypes. Scale bar= 250 μm. Quantification of the body length of a-b (mouth to end of tail through center of ear vesicle) in sibling and vhl mutant embryos at 48 hpf (A, right panel). And quantification of the head length of a-b (mouth to center of ear vesicle) and trunk length of b-c (center of ear vesicle to end of tail) in sibling and vhl mutant embryos at 96 hpf (B, right panel). The total embryo numbers are given below the X-axis. Values are mean ± S.D. Unpaired t-test. ns, not significant; ** p < 0.01 (TIF 6504 KB) [file 18_2025_5852_MOESM10_ESM.tif]

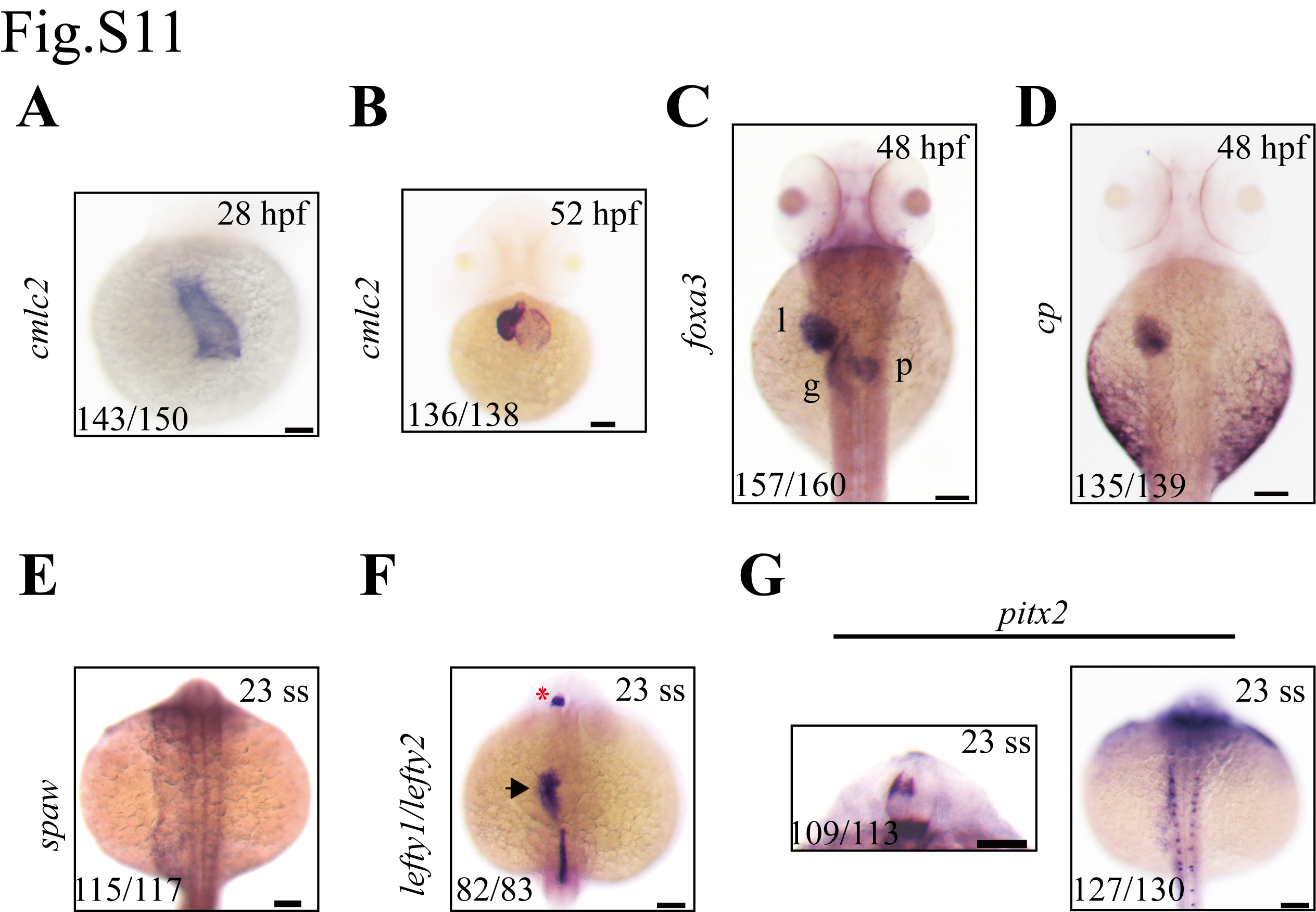

Supplement: Supplementary file 11 — Supplementary file11 Depletion of pVhl had little effect on left-right asymmetric development (A-G) Offspring embryos of heterozygous vhl mutants are examined for cmlc2 expression at 28 hpf (A) and 52 hpf (B), foxa3 expression at 48 hpf (C), cp expression at 48 hpf (D), spaw expression at the 23-somite stage (E), lefty1/lefty2 expression at the 23-somite stage (F), pitx2 expression in head (left) and LPM (right) at the 23-somite stage (G). Embryos are shown in ventral (A, B) or dorsal view (C-G) with anterior side upward. The red asterisk indicates the expression of lefty1 in the diencephalon, and the black arrow indicates the expression of lefty2 in heart field (F). l, liver; p, pancreas; g, gut. Embryos are from at least three pairs of adult fish. Scale bar = 100 μm (TIF 13002 KB) [file 18_2025_5852_MOESM11_ESM.tif]
