# Supplementary material for: pVHL regulates protein stability of the TCF/LEF transcription factor family via ubiquitin-independent proteasomal degradation
Source: Cell Mol Life Sci. 2025 Sep 4;82(1):335. doi: 10.1007/s00018-025-05852-0 (PMC12411348; doi:10.1007/s00018-025-05852-0)
Supplement: Supplementary file 12 — Supplementary file12 (PDF 117 KB) [file 18_2025_5852_MOESM12_ESM.pdf]

Supplementary file12. List of primers and sequence information.

name	Sequence (5'-3')
TCF7-qF	GTTCACCCACCCATCCTTGATGC
TCF7-qR	CAGCCTGGGTATAGCTGCATGTG
TCF7L1-qF	GTCAACGAGTCGGAGAACCA
TCF7L1-qR	TCTCACTTCGGCGAAATAGTC
TCF7L2-qF	CCTCGGCAGAGAGGGATTTAGCTG
TCF7L2-qR	GAGCCCTCCATCTTGCCTCTTG
HIF-1α-qF	TATGAGCCAGAAGAACTTTTAGGC
HIF-1α-qR	CACCTCTTTTGGCAAGCATCCTG
NKD1-qF	GCCTTGGTGGTGTATGAGAG
NKD1-qR	CTGGTAGAAGTGGTGGTAATGG
AXIN2-qF	CTGGCTTTGGTGAACTGTTG
AXIN2-qR	AGTTGCTCACAGCCAAGACA
CCND1-qF	CCATCCAGTGGAGGTTTGTC
CCND1-qR	AGCGTATCGTAGGAGTGGGA
β-ACTIN-qF	ACCCTGAAGTACCCCATCGAG
β-ACTIN-qR	GGATAGCACAGCCTGGATAGCA
AXIN2-WRE-F	CTGGAGCCGGCTGCGCTTTGATAA
AXIN2-WRE-R	CGGCCCCGAAATCCATCGCTCTGA
NKD1-WRE-F	TGCCAGGACGAGCGTAACAC
NKD1-WRE-R	GGATTCCACCCCACCTTTG
VHL-CAS9-Target-F	GGACGAAACACCGATACGGGCAGCACGACGCGGTTTTAGAGCTA
VHL-CAS9-Target-R	TTTCTAGCTCTAAAACCGCGTCGTGCTGCCCGTATCGGTGTTTC
VHL-CAS9-Test-F	GCGTTCCATCCTCTACCG
VHL-CAS9-Test-R	GGGCTTCAGACCGTGCTAT
HIF1-β-CAS9-Target-F	GGACGAAACACCGTGAAATTGAACGGCGGCGAGTTTTAGAGCTA
HIF1-β-CAS9-Target-R	TTTCTAGCTCTAAAACTCGCCGCCGTTCAATTTCACGGTGTTTC
HIF1-β-CAS9-Test-F	CAAGTAATCCACCTGCCTCCATCTC
HIF1-β-CAS9-Test-R	TTCCCCGCAAGGACTTCAT
