# Supplementary material for: pVHL regulates protein stability of the TCF/LEF transcription factor family via ubiquitin-independent proteasomal degradation
Source: Cell Mol Life Sci. 2025 Sep 4;82(1):335. doi: 10.1007/s00018-025-05852-0 (PMC12411348; doi:10.1007/s00018-025-05852-0)
Supplement: Supplementary file 13 — Supplementary file13 (PDF 144 KB) [file 18_2025_5852_MOESM13_ESM.pdf]

Supplementary file13. List of antibodies.

Antibody	Source	Host	Identifier	Dilution Rate
TCF7	Cell Signaling Technology	Rabbit	2203	1:1000 (WB)
TCF7L1	Cell Signaling Technology	Rabbit	2883	1:1000 (WB)
TCF7L2	Cell Signaling Technology	Rabbit	2569	1:1000 (WB)<br>1:50 (CHIP)
TCF7L1/TCF7L2	Abcam	Mouse	ab12065	1:200 (IF)
pVHL	Cell Signaling Technology	Rabbit	68547	1:1000 (WB)
pVHL	Santa Cruz Biotechnology	Mouse	sc-135657	1:1000 (WB)
proteasome 19S S5A	Abcam	Rabbit	ab137109	1:1000 (WB)
PSMA4	zenbio	Rabbit	R25489	1:1000 (WB)
HIF-1α	BD Bioscience,	Mouse	610958	1:1000 (WB)
HIF-2α	Novus Biologicals	Rabbit	NB 100-122	1:1000 (WB)
HIF-1β	ABclonal	Rabbit	A19532	1:1000 (WB)
β-catenin (44C6)	Abmart	Mouse	M24002	1:1000 (WB)
non-p-β-catenin	Cell Signaling Technology	Rabbit	8814	1:1000 (WB)
ELOC	Affinity Biosciences	Rabbit	DF2373	1:1000 (WB)
Ubiquitin	Santa Cruz Biotechnology	Mouse	SC-8017	1:1000 (WB)
Flag	Sigma	Mouse	F1804	1:1000 (WB)<br>1:500 (IF)
Myc	Santa Cruz Biotechnology	Mouse	sc-40	1:1000 (WB)
HA	Cell Signaling Technology	Rabbit	3724S	1:1000 (WB);<br>1:500 (IF)
GFP	Abmart	Rabbit	P30010L	1:1000 (WB)
Histone H3.1	Abmart	Rabbit	P30266	1:4000 (WB)
GAPDH	BBI	Rabbit	D110016	1:4000 (WB)
β-ACTIN	Absin	Rabbit	ABS132001	1:4000 (WB)
HRP-labeled Goat Anti-Mouse IgG(H+L)	Beyotime	Goat	A0216	1:4000 (WB)
HRP-labeled Goat Anti-Rabbit IgG(H+L)	Beyotime	Goat	A0208	1:4000 (WB)
AF488-labeled Goat	Beyotime	Goat	A0423	1:500 (IF)

Anti-Rabbit IgG (H+L)				
Cy3-labeled Goat Anti-Mouse IgG (H+L)	Beyotime	Goat	A0521	1:500 (IF)
